# Supplementary material for: Striking Variability in the Post-Reproductive Movements of Spanish Red Kites (Milvus milvus): Three Strategies, Sex Differences, and Changes over Time
Source: Animals (Basel). 2022 Oct 25;12(21):2930. doi: 10.3390/ani12212930 (PMC9655320; doi:10.3390/ani12212930)
Supplement: Supplementary file 1 [file animals-12-02930-s001.zip › animals-1921937-supplementary.pdf]

ELECTRONIC SUPPLEMENTARY MATERIALS

Table S1. Metadata and parameters of the 47 tagged Red Kites. For Sex, F = female and M = male. For Strategy, Sed = sedentary, Mig = migratory, and SedMov = sedentary with post-reproductive movements. The dash (-) indicates that the information is not available.

*From July to February

ID	Sex	Spent the entire wintering season outside breeding area	Strategy (cualitative classification)	Nest Latitude	Maximum latitudinal displacement from the nest (º)	Maximum Distance to nest (km)	Mean Distance to Nest (km)	Days outside the breeding área (> 50 km away)	Period*
Araba 01	F	NO	SedMov	42.730	2.2	264	10.5 ± 41.9	7	2018/19
		NO	SedMov		1.9	269	7.1 ± 29.7	5	2019/20
Araba 02	F	NO	Sed	42.689	0.2	39.9	7.6 ± 5.7	0	2020/21
Araba 09	F	NO	Sed	42.922	0.3	35.9	6.5 ± 6.8	0	2019/20
		NO	Sed		0.3	16.4	4.9 ± 3.9	0	2020/21
Araba 10	F	NO	Sed	42.845	0.1	8.1	2.2 ± 3.3	0	2019/20
		NO	Sed		0.1	26.7	1.3 ± 1.97	0	2020/21
Avila 01	F	NO	Sed	40.697	0.1	7.29	1.4 ± 1.6	0	2015/16
		NO	Sed		0.1	10.2	1.3 ± 1.5	0	2016/17
		NO	Sed		0.1	12.9	4.7 ± 4.5	0	2017/18
		NO	Sed		0.2	18.8	0.9 ± 1.3	0	2018/19
		NO	Sed		0.1	5.6	1.0 ± 1.1	0	2019/20
		NO	Sed		0.1	3.8	0.8 ± 0.6	0	2020/21
Avila 02	F	NO	Sed	40.867	0.2	14.3	5.7 ± 3.4	0	2015/16
Avila 03	M	NO	Sed	40.783	0.2	18.8	6.2 ± 5.1	0	2015/16
		NO	Sed		0.3	18.7	5.3 ± 4.2	0	2016/17
		NO	Sed		0.3	20.8	5.3 ± 5.1	0	2017/18
		NO	Sed		0.3	18.0	2.7 ± 3.0	0	2018/19
Burgos 01	F	NO	SedMov	42.047	1.3	148.6	4.7 ± 12.9	3	2016/17
		NO	Sed		0.2	27.5	3.7 ± 6.4	0	2017/18
Caceres 01	F	NO	Sed	39.972	0.1	11.3	2.0 ± 2.9	0	2014/15
		NO	Sed		0.1	12.2	4.4 ± 4.3	0	2015/16
Caceres 02	F	NO	SedMov	39.901	1.7	175.3	11.9 ± 31.8	175	2017/18
		NO	SedMov		1.2	142.5	27.7 ± 44.5	157	2018/19
Guipuzcoa 01	M	NO	Sed	43.090	0.2	19.9	1.2 ± 1.5	0	2015/16
		NO	Sed		0.1	12.2	1.2 ± 1.1	0	2016/17
		NO	Sed		0.1	11.5	1.2 ± 1.1	0	2017/18

		NO	Sed		0.1	8.2	1.1 ± 0.7	0	2018/19
Guipuzcoa 02	F	NO	Sed	43.179	0.1	5.6	1.5 ± 1.1	0	2015/16
Guipuzcoa 04	F	YES	Mig	41.001	4.6	429.6	233 ± 97	113	2019/20
		NO	SedMov	43.134	4	589.2	14.3 ± 70.1	7	2020/21
Guipuzcoa 05	M	NO	Sed	43.165	0.1	7.4	1.9 ± 1.9	0	2019/20
		NO	Sed		0.1	16.1	1.9 ± 2.2	0	2020/21
Huesca 06	M	NO	Sed	42.212	0.3	33.7	8.2 ± 7.5	0	2015/16
		NO	Sed		0.2	38.7	7.7 ± 7.0	0	2016/17
		NO	Sed		0.3	46.6	7.2 ± 7.9	0	2017/18
		NO	Sed		0.3	45.3	5.9 ± 7.0	0	2018/19
		NO	Sed		0.3	42.8	9.2 ± 8.5	0	2019/20
Leon 02	F	NO	Sed	42.248	0.1	8.1	1.9 ± 1.8	0	2017/18
		NO	SedMov		1.3	147.6	13.8 ± 38.3	19	2018/19
		NO	SedMov		1.2	129.4	10.9 ± 33.5	18	2019/20
		NO	Sed		0.1	7.4	1.0 ± 1.0	0	2020/21
Madrid 02	F	NO	Sed	40.924	0.5	48.8	7.9 ± 11.7	0	2013/14
		NO	Sed		0.5	49.2	3.9 ± 7.5	0	2014/15
		NO	Sed		0.5	40.4	6.4 ± 7.3	0	2015/16
Madrid 03	M	NO	SedMov	40.407	0.4	83.8	13.6 ± 11.5	9	2014/15
Madrid 04	F	NO	Sed	40.718	0.1	37.2	3.5 ± 5.9	0	2014/15
		NO	Sed		0.1	18.5	3.2 ± 3.6	0	2015/16
		NO	Sed		0.1	12.4	2.8 ± 3.4	0	2016/17
		NO	Sed		0.1	18.2	2.5 ± 4.0	0	2017/18
		NO	Sed		0.1	14.2	1.5 ± 2.5	0	2018/19
		NO	Sed		0.1	17.9	2.0 ± 2.3	0	2019/20
		NO	Sed		0.1	14.2	1.7 ± 3.3	0	2020/21
Palencia 01	F	NO	SedMov	42.899	2.2	230.3	98.2 ± 61.3	216	2016/17
		NO	SedMov		1.2	128.5	15.5 ± 21.8	44	2017/18
Salamanca 01	F	NO	Sed	40.877	0.4	44.5	2.0 ± 3.0	0	2017/18
		NO	Sed		0.2	23.5	1.2 ± 1.6	0	2018/19
		NO	Sed		0.2	34.9	2.9 ± 5.1	0	2019/20
		NO	Sed		0.1	25.4	1.8 ± 2.3	0	2020/21
Salamanca 02	F	NO	SedMov	40.924	0.4	76.6	6.3 ± 9.1	5	2017/18
Segovia 04	F	NO	Sed	41.031	0.1	9.9	1.4 ± 1.7	0	2015/16
		NO	Sed		0.1	8.9	1.4 ± 1.6	0	2016/17
		NO	Sed		0.1	9.7	1.2 ± 1.6	0	2017/18
		NO	Sed		0.1	8.6	1.0 ± 1.2	0	2018/19
		NO	Sed		0.1	6.8	0.8 ± 0.5	0	2019/20
Segovia	F	NO	Sed	41.244	0.1	14.2	1.0 ± 1.3	0	2015/16

05									
Soria 01	F	YES	Mig	41.986	5.3	639.0	235 ± 263	116	2015/16
		YES	Mig		5.3	599.2	260 ± 237	162	2016/17
Soria 02	M	NO	Sed	41.938	0.3	40.5	2.9 ± 5.7	0	2015/16
		NO	Sed		0.3	35.8	2.4 ± 4.9	0	2016/17
		NO	Sed		0.2	34.6	2.3 ± 5.3	0	2017/18
		NO	Sed		0.2	42.0	2.1 ± 4.8	0	2018/19
		NO	Sed		0.2	36.0	3.4 ± 7.4	0	2019/20
		NO	Sed		0.2	42.3	3.3 ± 7.4	0	2020/21
Soria 03	F	YES	Mig	41.994	2.3	318.6	144 ± 145	119	2015/16
Soria 04	F	NO	Sed	42.553	0.1	13.2	1.4 ± 1.0	0	2018/19
		NO	Sed		0.1	8.0	1.3 ± 0.6	0	2019/20
		NO	Sed		0.1	3.8	1.4 ± 0.5	0	2020/21
Soria 05	F	YES	Mig	41.965	1.3	373.3	51.2 ± 108	90	2016/17
		NO	SedMov		1.4	354.9	25.2 ± 74.8	18	2017/18
		NO	SedMov		1.1	270.9	22.6 ± 63.2	12	2018/19
		NO	SedMov		0.4	74.1	28.3 ± 79.5	3	2019/20
		NO	SedMov		1.1	368.0	25.2 ± 77.8	21	2020/21
Toledo 03	M	YES	Mig	39.608	4.6	780.9	189 ± 302	78	2017/18
		NO	SedMov		0.5	86.0	7.7 ± 15.4	6	2018/19
		NO	Sed		0.2	19.6	3.4 ± 3.9	0	2019/20
		YES	Mig		4.6	774.1	373 ± 365	64	2020/21
Valladolid 02	F	NO	SedMov	41.441	0.5	79.5	25.4 ± 30.7	93	2015/16
		NO	SedMov		0.5	78.8	24.0 ± 30.6	71	2016/17
Zamora 01	F	NO	SedMov	41.324	0.2	188.1	16.3 ± 37.1	17	2015/16
		NO	SedMov		0.1	141.4	22.4 ± 46.2	28	2016/17
		NO	SedMov		0.2	141.5	28.9 ± 48.3	28	2017/18
Zaragoza 02	F	NO	SedMov	42.33	0.7	177.7	4.0 ± 10.6	30	2016/17
		NO	Sed		0.3	24.4	1.5 ± 2.4	0	2017/18
		NO	Sed		0.3	31.1	3.7 ± 5.5	0	2018/19
		NO	Sed		0.3	19.9	2.4 ± 3.6	0	2019/20
		NO	Sed		0.1	19.6	2.3 ± 3.7	0	2020/21
Madrid 17	M	NO	Sed	40.690	0.1	24.4	1.2 ± 2.0	0	2019/20
		NO	Sed		0.1	6.0	1.1 ± 1.3	0	2020/21
Madrid 11	F	NO	SedMov	40.667	0.9	89.0	30.7 ± 33.5	71	2018/19
Madrid 19	F	NO	Sed	40.709	0.1	23.4	3.2 ± 5.2	0	2020/21
Mad	M	YES	Mig	40.714	1	270.1	119 ± 105	149	2014/15

rid 01		YES	Mig		1	249.4	95.9 ± 114	96	2015/16
		YES	Mig		1	264.5	105 ± 106	126	2016/17
		YES	Mig		1	249.2	76.8 ± 108	76	2017/18
		NO	Sed		0.1	15.7	2.2 ± 3.7	0	2018/19
		NO	Sed		0.1	12.0	2.3 ± 3.8	0	2019/20
		NO	Sed		0.1	11.2	3.0 ± 4.3	0	2020/21
Madrid 07	M	NO	Sed	40.655	0.1	11.7	1.0 ± 1.1	0	2017/18
		NO	Sed		0.1	3.4	0.8 ± 0.5	0	2018/19
Madrid 16	F	NO	SedMov	40.529	0.6	217.0	32.7 ± 69.7	28	2019/20
		NO	SedMov		0.6	214.3	69.8 ± 91.4	87	2020/21
Madrid 14	F	NO	Sed	39.674	0.1	18.0	5.7 ± 6.5	0	2017/18
		NO	Sed		0.1	17.9	2.6 ± 4.4	0	2018/19
		NO	Sed		0.2	25.2	3.9 ± 5.0	0	2019/20
		NO	Sed		0.1	20.4	3.9 ± 3.9	0	2020/21
Madrid 20	F	NO	Sed	40.669	0.2	48.9	11.2 ± 7.0	0	2020/21
Madrid-Fleybea	F	NO	SedMov	38.172	0.9	130.2	23.0 ± 44.1	42	2016/17
		NO	Sed		0.1	11.0	2.8 ± 1.4	0	2017/18
		NO	Sed		0.1	10.2	3.1 ± 1.5	0	2018/19
		NO	Sed		0.1	10.0	3.6 ± 1.6	0	2019/20
Madrid-Jara	F	NO	Sed	40.181	0.2	24.7	14.9 ± 8.7	0	2015/16
		NO	Sed		0.2	24.6	17.5 ± 7.9	0	2016/17
		NO	Sed		0.2	25.3	17.4 ± 7.1	0	2017/18
		NO	Sed		0.2	24.7	18.0 ± 7.1	0	2018/19
		NO	Sed		0.2	25.8	15.7 ± 8.8	0	2019/20
Madrid-Roman	M	NO	Sed	40.485	0.3	35.2	3.4 ± 6.6	0	2020/21
Madrid-Roncesvalles	-	NO	SedMov	41.332	0.7	77.9	12.7 ± 23.1	23	2019/20
		NO	Sed		0.1	19.7	4.1 ± 3.2	0	2020/21
Madrid-Salvia	F	YES	Mig	40.211	2.2	385.4	174 ± 143	158	2019/20
		YES	Mig		2.2	324.7	134 ± 79.1	85	2020/21
Madrid-Sucesso	M	NO	Sed	40.678	0.2	8.0	1.9 ± 1.8	0	2013/14
		NO	Sed		0.1	6.1	1.8 ± 1.3	0	2014/15
		NO	Sed		0.1	6.5	2.1 ± 1.5	0	2015/16
		NO	Sed		0.1	7.0	2.1 ± 1.4	0	2016/17
		NO	Sed		0.1	6.2	1.7 ± 0.4	0	2018/19

Table S2. Importance of the components in the PCA analyses.

	Component 1	Component 2	Component 3	Component 4
**Standard deviation**	1.82	0.67	0.41	0.26
**Proportion of variance**	0.82	0.11	0.04	0.02
**Cumulative Proportion**	0.82	0.94	0.98	1

Table S3. Results of the Linear Mixed Models (*LMM*). "Strategy" (migratory, sedentary with post-reproductive movements, and sedentary) was considered as fixed effect. "Period" and "individual" were considered as random effects, and "period" was nested inside "individual". Estimates, standard error (SE), degrees of freedom (df), t value and *p* value are shown. Sed = sedentary; Mig= migrator. N = 136.

Variable	Factor	Estimates	SE	df	t value	*p*
**Latitudinal displacement**	Intercept	1.27	0.11	55.17	11.76	**<0.0001**
	Strategy (Mig)	1.2	0.11	119.04	10.86	**<0.0001**
	Strategy (Sed)	-0.99	0.09	131.48	-11.57	**<0.0001**
**Days outside breeding area**	Intercept	52.2	4.44	55.41	11.75	**<0.0001**
	Strategy (Mig)	61.25	5.05	126.32	12.12	**<0.0001**
	Strategy (Sed)	-48.15	3.83	132.68	-12.56	**<0.0001**
**Mean distance to nest**	Intercept	66.05	3.74	69.14	17.66	**<0.0001**
	Strategy (Mig)	104.15	5.82	122.22	17.89	**<0.0001**
	Strategy (Sed)	-61.09	4.02	98.13	-15.21	**<0.0001**
**Maximum distance to nest**	Intercept	206.65	12.24	28.09	16.88	**<0.0001**
	Strategy (Mig)	217.79	16.27	128.28	13.39	**<0.0001**
	Strategy (Sed)	-183.78	11.63	101.97	-15.8	**<0.0001**

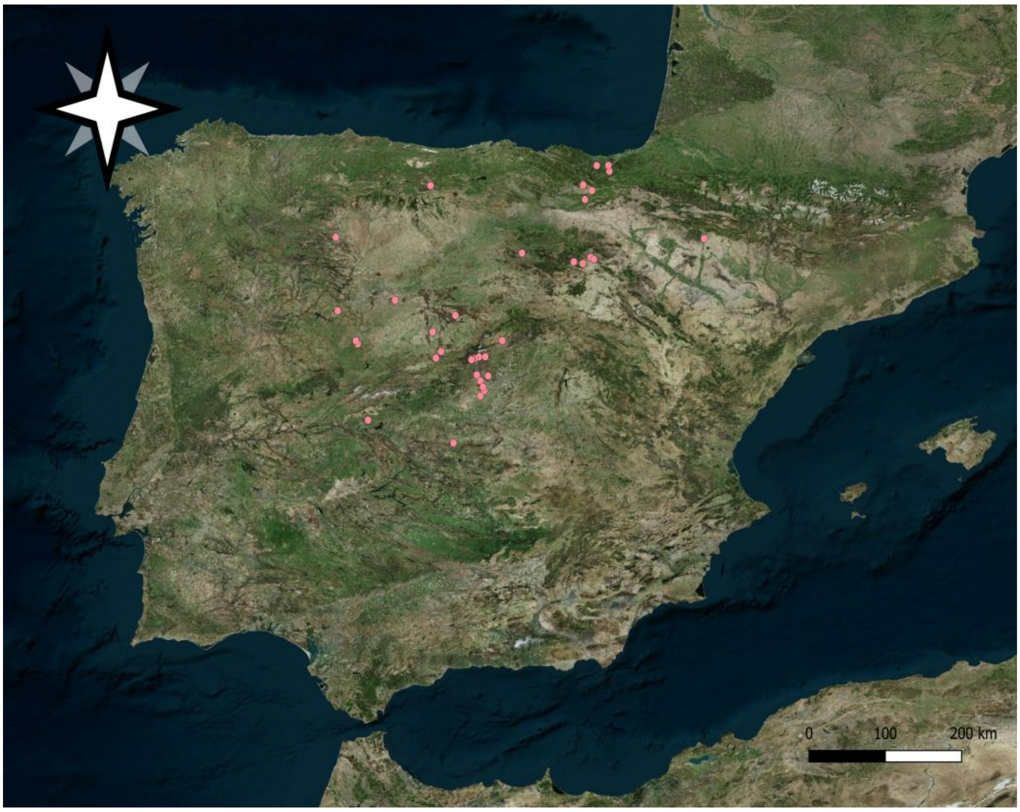

Figure S1. Tagging points (pink circles) of the individuals.

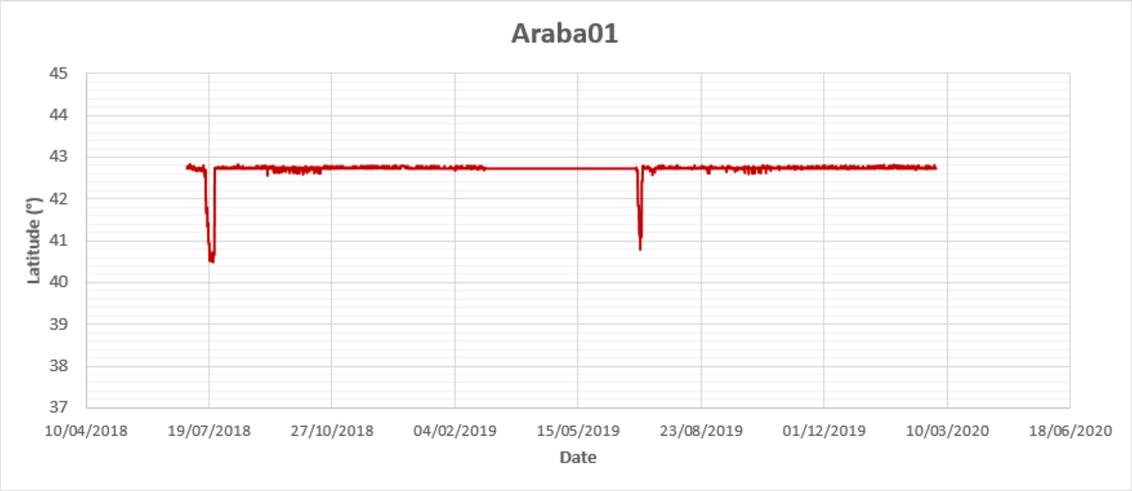

Araba01
45
44
43
42
41
40
39
38
37
Latitude (°)
10/04/2018
19/07/2018
27/10/2018
04/02/2019
15/05/2019
23/08/2019
01/12/2019
10/03/2020
18/06/2020
Date

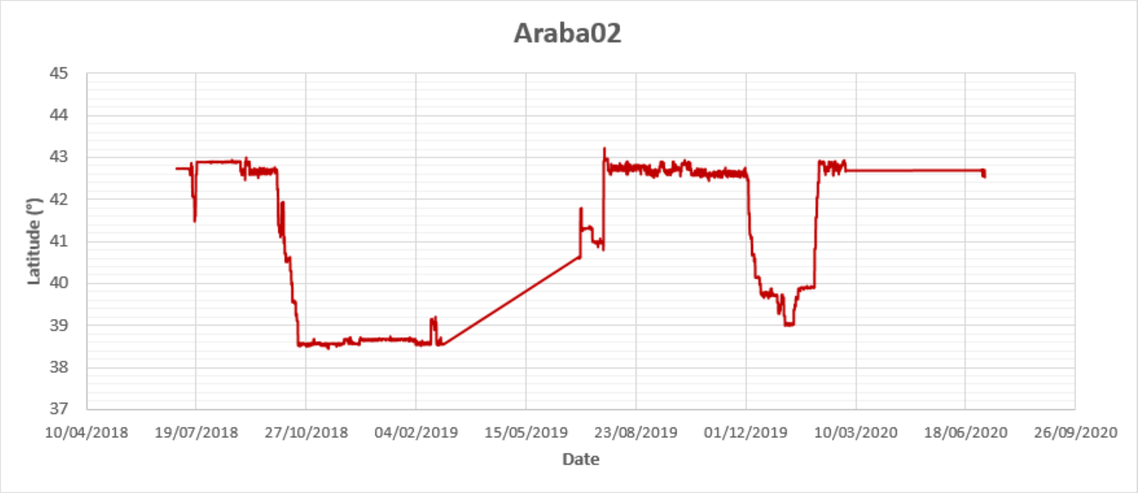

Araba02
45
44
43
42
41
40
39
38
37
Latitude (°)
10/04/2018
19/07/2018
27/10/2018
04/02/2019
15/05/2019
23/08/2019
01/12/2019
10/03/2020
18/06/2020
26/09/2020
Date

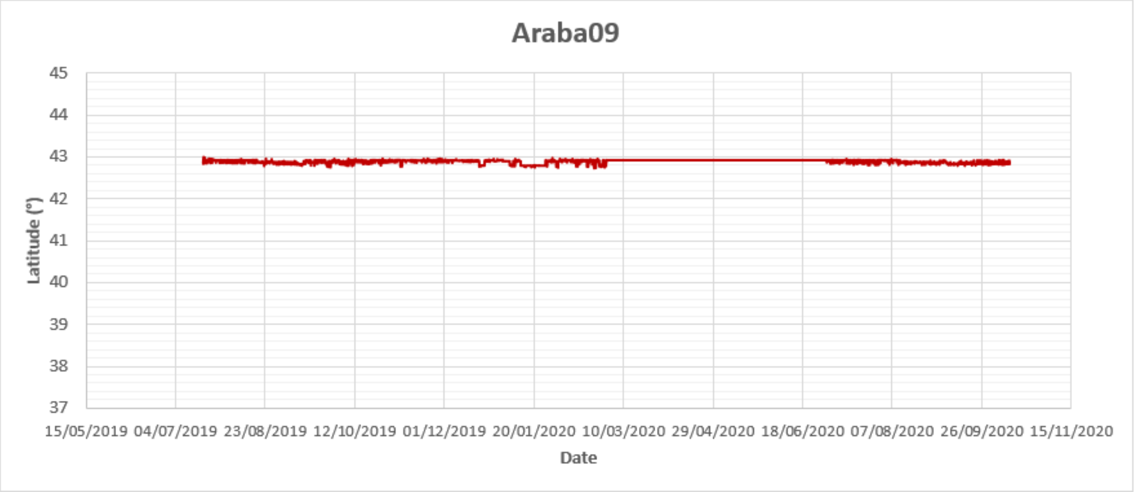

Araba09
45
44
43
42
41
40
39
38
37
Latitude (°)
15/05/2019
04/07/2019
23/08/2019
12/10/2019
01/12/2019
20/01/2020
10/03/2020
29/04/2020
18/06/2020
07/08/2020
26/09/2020
15/11/2020
Date

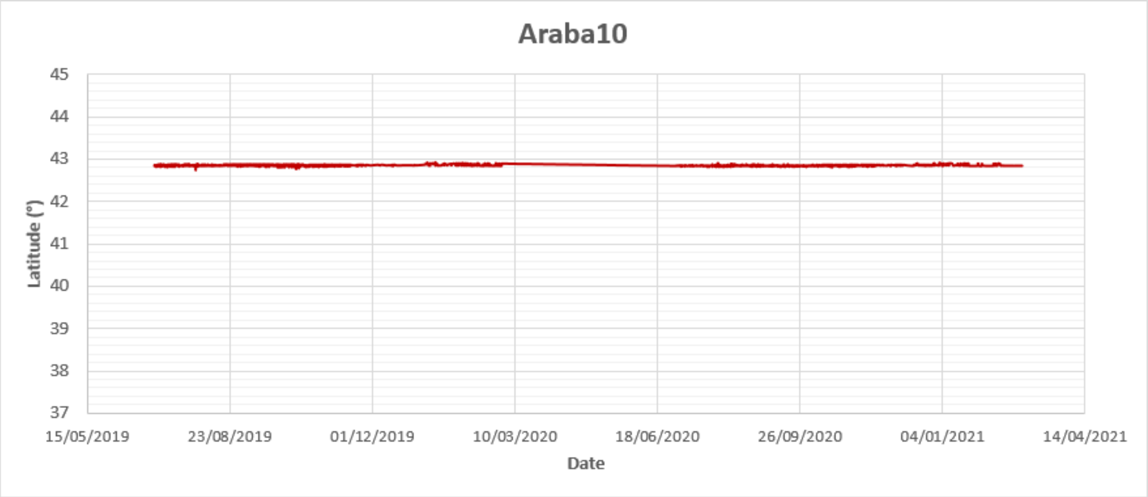
Araba10
45
44
43
42
41
40
39
38
37
Latitude (°)
15/05/2019
23/08/2019
01/12/2019
10/03/2020
18/06/2020
26/09/2020
04/01/2021
14/04/2021
Date

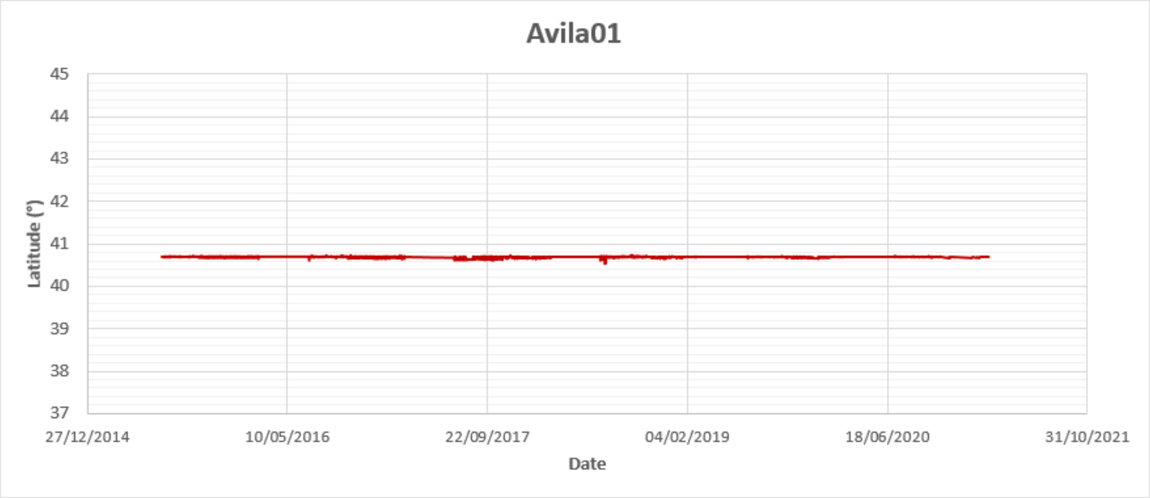
Avila01
45
44
43
42
41
40
39
38
37
Latitude (°)
27/12/2014
10/05/2016
22/09/2017
04/02/2019
18/06/2020
31/10/2021
Date

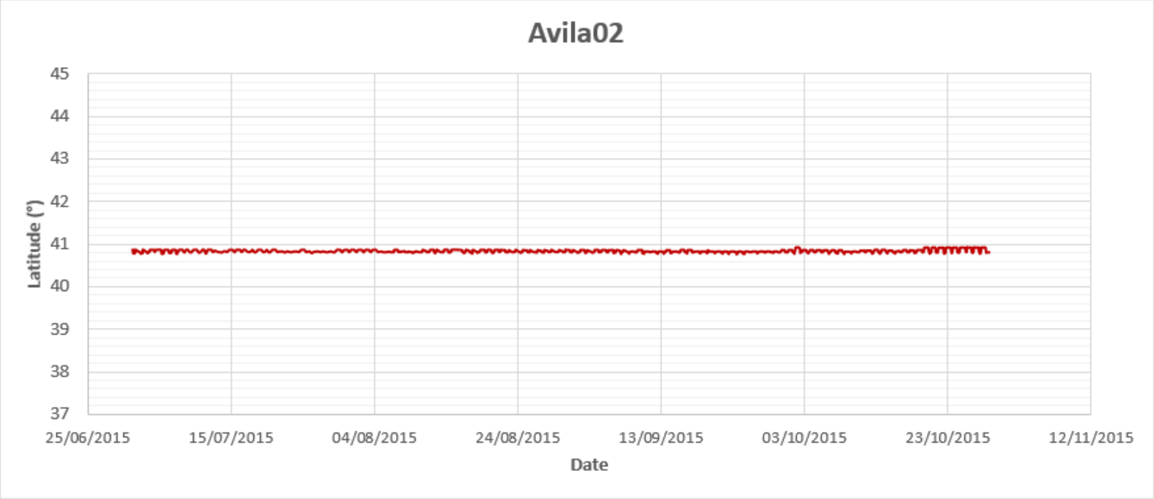
Avila02
45
44
43
42
41
40
39
38
37
Latitude (°)
25/06/2015
15/07/2015
04/08/2015
24/08/2015
13/09/2015
03/10/2015
23/10/2015
12/11/2015
Date

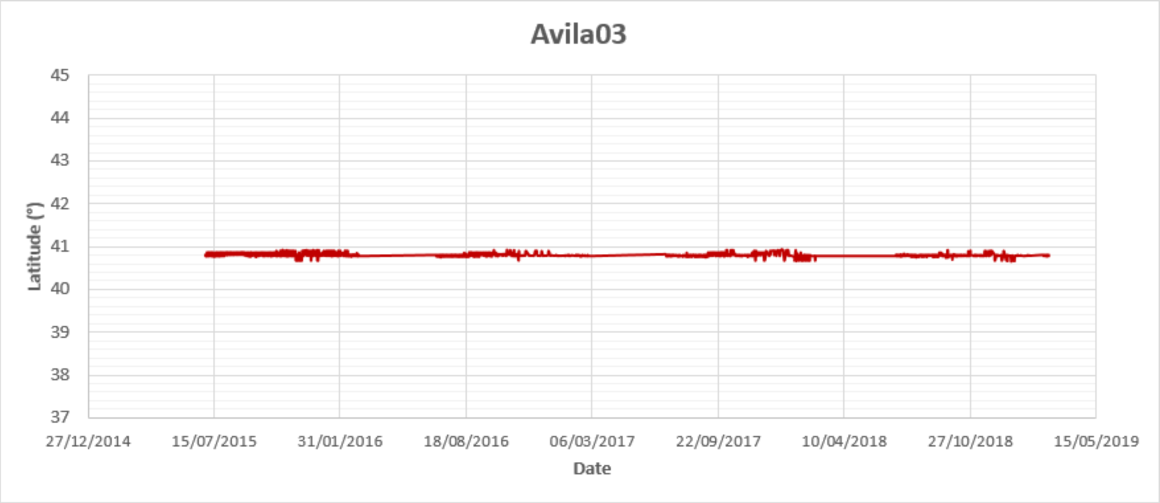
Avila03
Latitude (°)
45
44
43
42
41
40
39
38
37
27/12/2014
15/07/2015
31/01/2016
18/08/2016
06/03/2017
22/09/2017
10/04/2018
27/10/2018
15/05/2019
Date

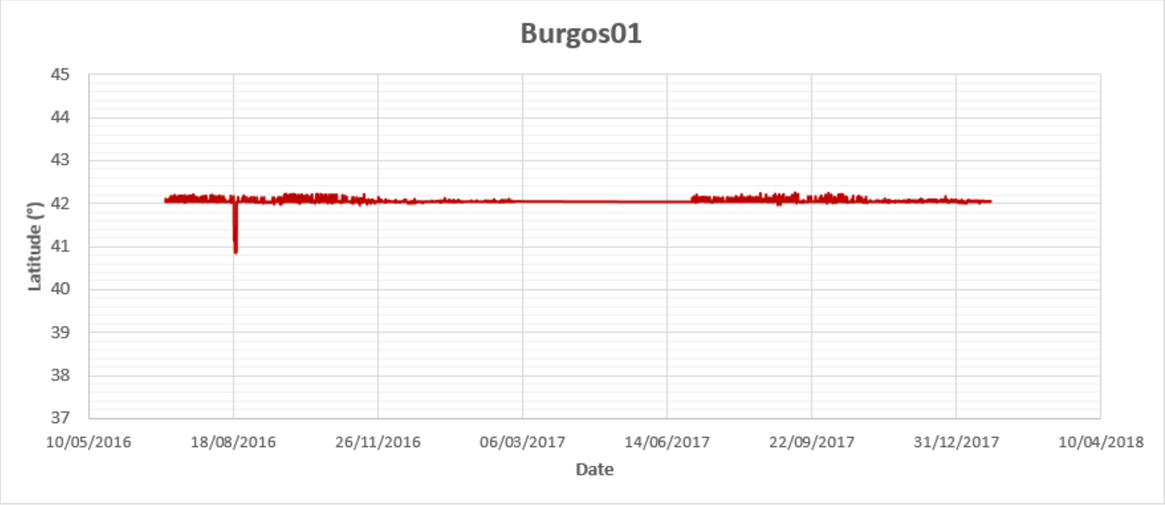
Burgos01
Latitude (°)
45
44
43
42
41
40
39
38
37
10/05/2016
18/08/2016
26/11/2016
06/03/2017
14/06/2017
22/09/2017
31/12/2017
10/04/2018
Date

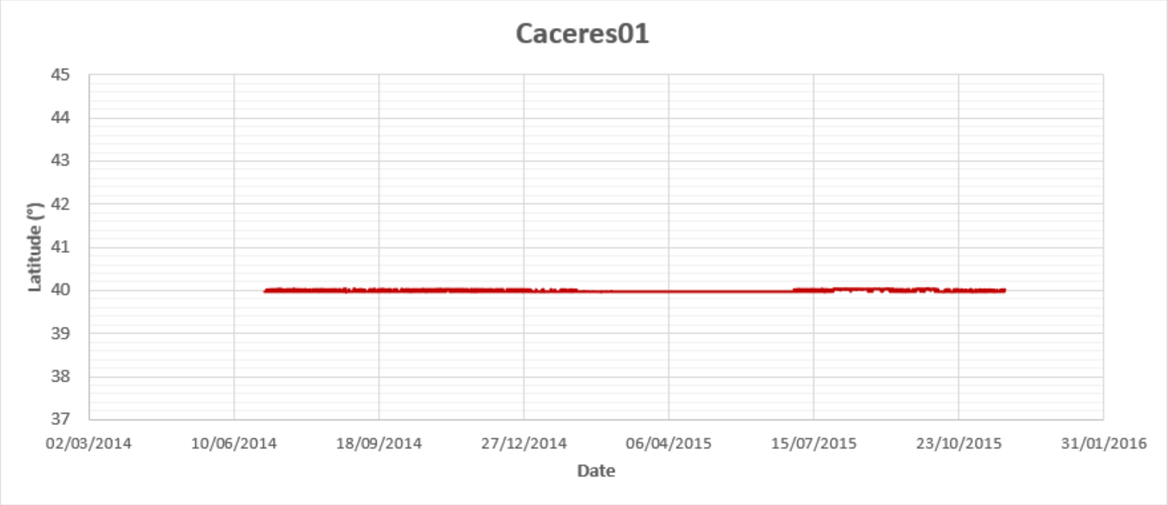
Caceres01
Latitude (°)
45
44
43
42
41
40
39
38
37
02/03/2014
10/06/2014
18/09/2014
27/12/2014
06/04/2015
15/07/2015
23/10/2015
31/01/2016
Date

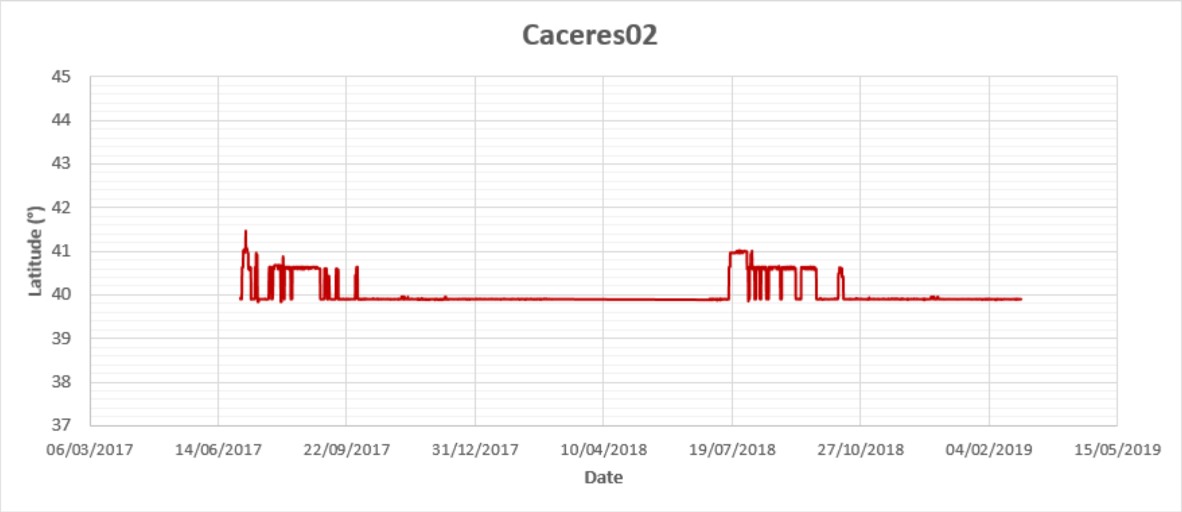
Caceres02
45
44
43
42
41
40
39
38
37
Latitude (°)
06/03/2017
14/06/2017
22/09/2017
31/12/2017
10/04/2018
19/07/2018
27/10/2018
04/02/2019
15/05/2019
Date

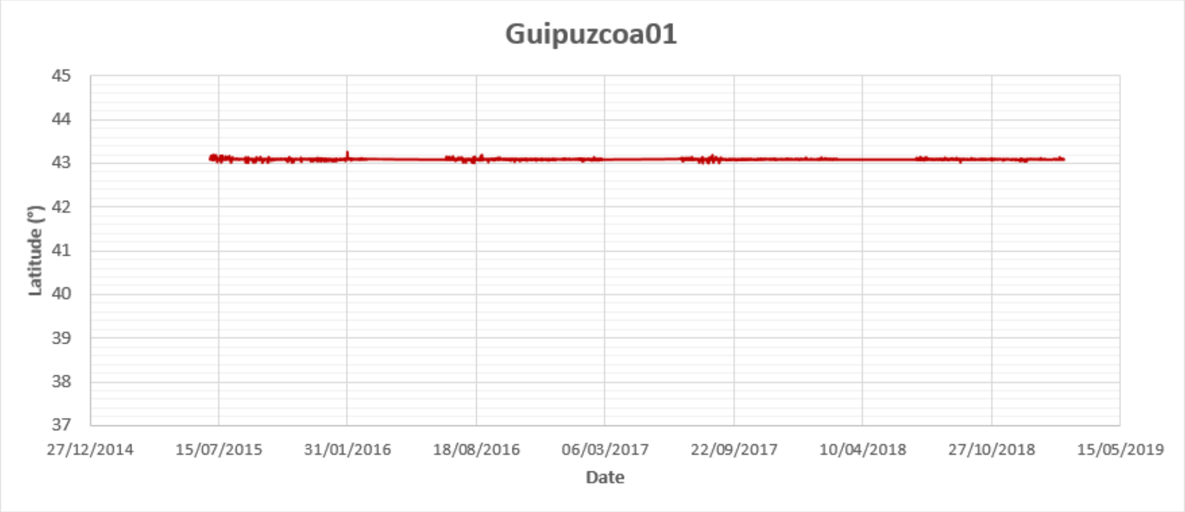
Guipuzcoa01
45
44
43
42
41
40
39
38
37
Latitude (°)
27/12/2014
15/07/2015
31/01/2016
18/08/2016
06/03/2017
22/09/2017
10/04/2018
27/10/2018
15/05/2019
Date

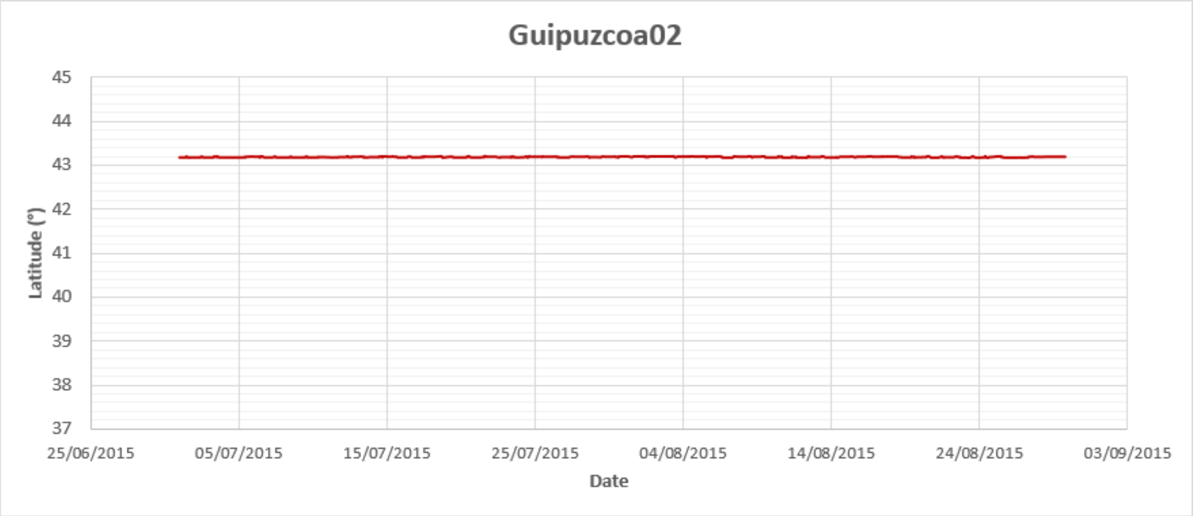
Guipuzcoa02
45
44
43
42
41
40
39
38
37
Latitude (°)
25/06/2015
05/07/2015
15/07/2015
25/07/2015
04/08/2015
14/08/2015
24/08/2015
03/09/2015
Date

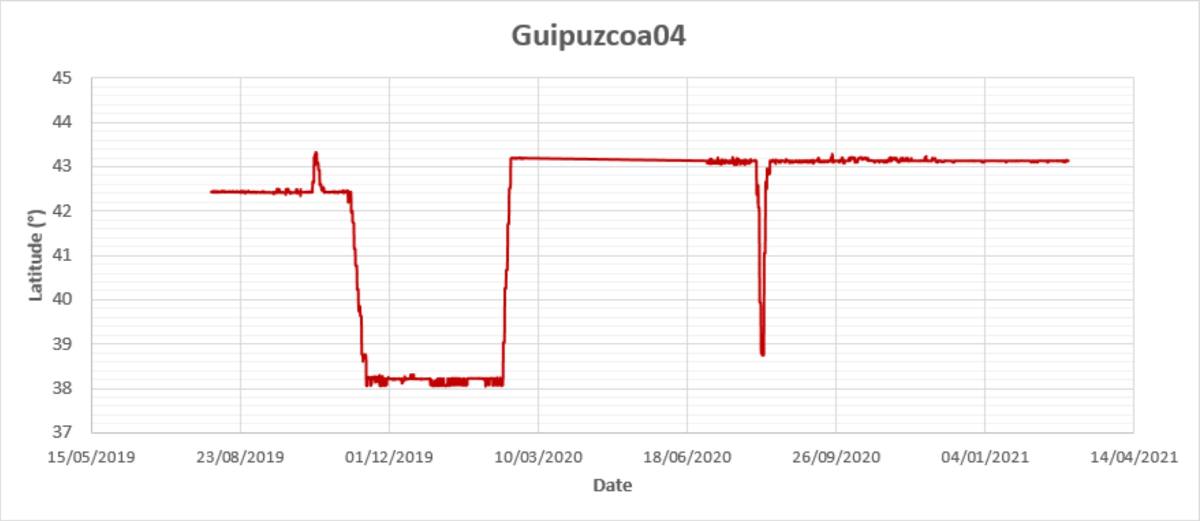
Guipuzcoa04
45
44
43
42
41
40
39
38
37
Latitude (°)
15/05/2019
23/08/2019
01/12/2019
10/03/2020
18/06/2020
26/09/2020
04/01/2021
14/04/2021
Date

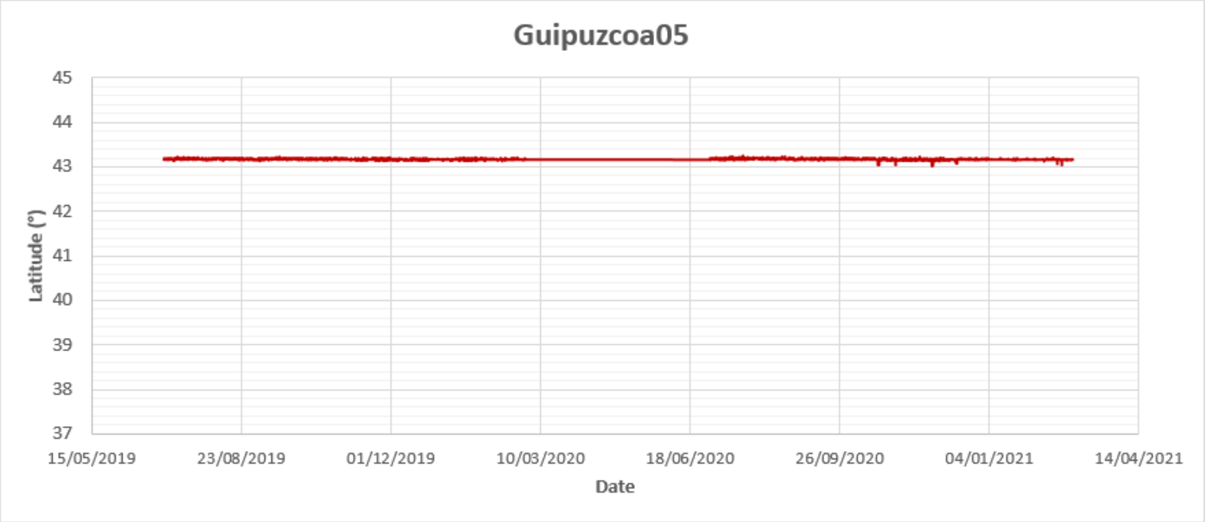
Guipuzcoa05
45
44
43
42
41
40
39
38
37
Latitude (°)
15/05/2019
23/08/2019
01/12/2019
10/03/2020
18/06/2020
26/09/2020
04/01/2021
14/04/2021
Date

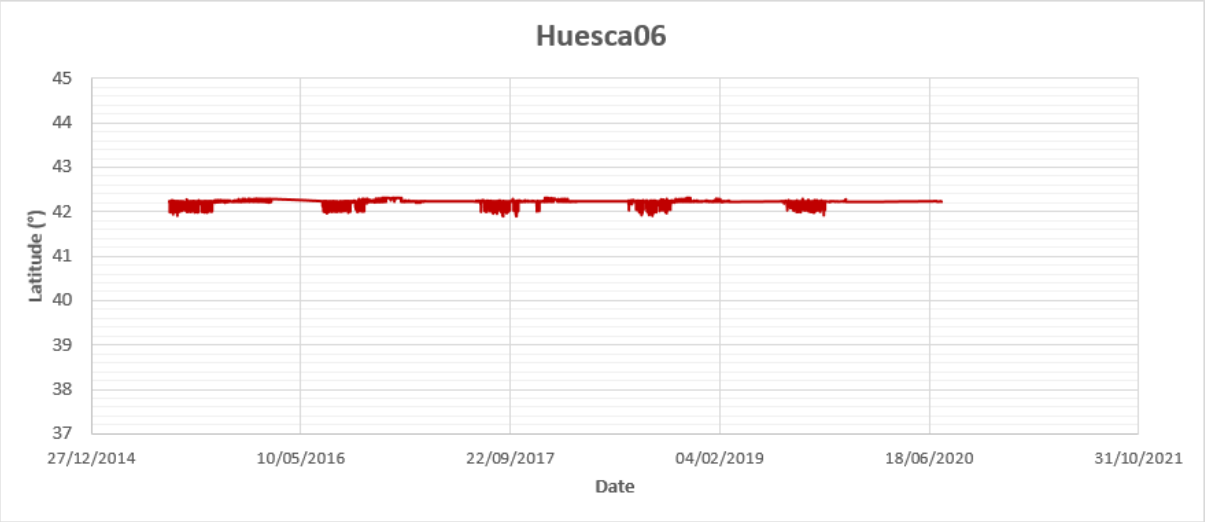
Huesca06
45
44
43
42
41
40
39
38
37
Latitude (°)
27/12/2014
10/05/2016
22/09/2017
04/02/2019
18/06/2020
31/10/2021
Date

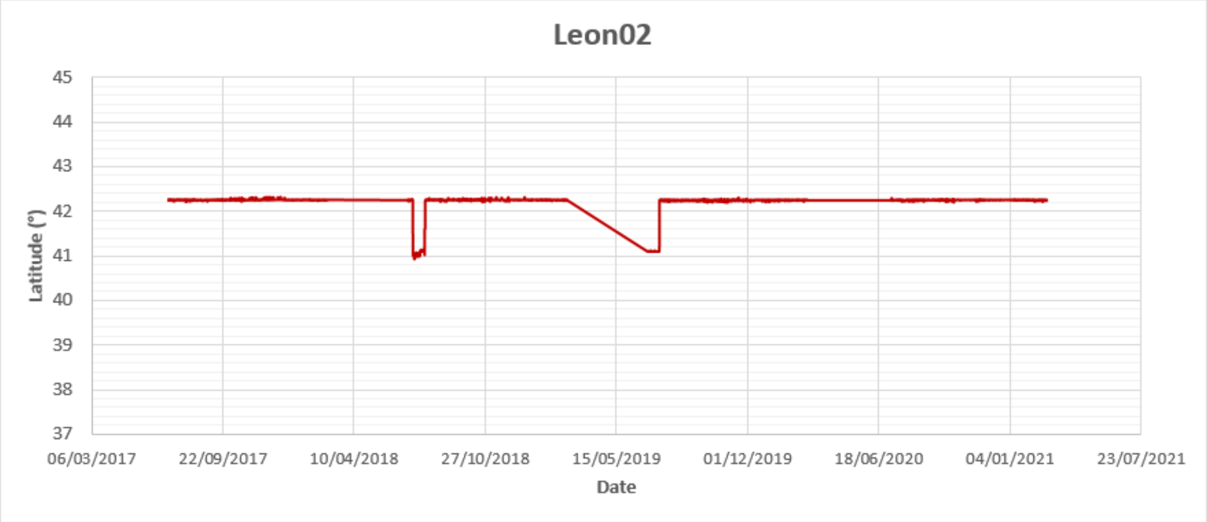
Leon02
45
44
43
42
41
40
39
38
37
Latitude (°)
06/03/2017
22/09/2017
10/04/2018
27/10/2018
15/05/2019
01/12/2019
18/06/2020
04/01/2021
23/07/2021
Date

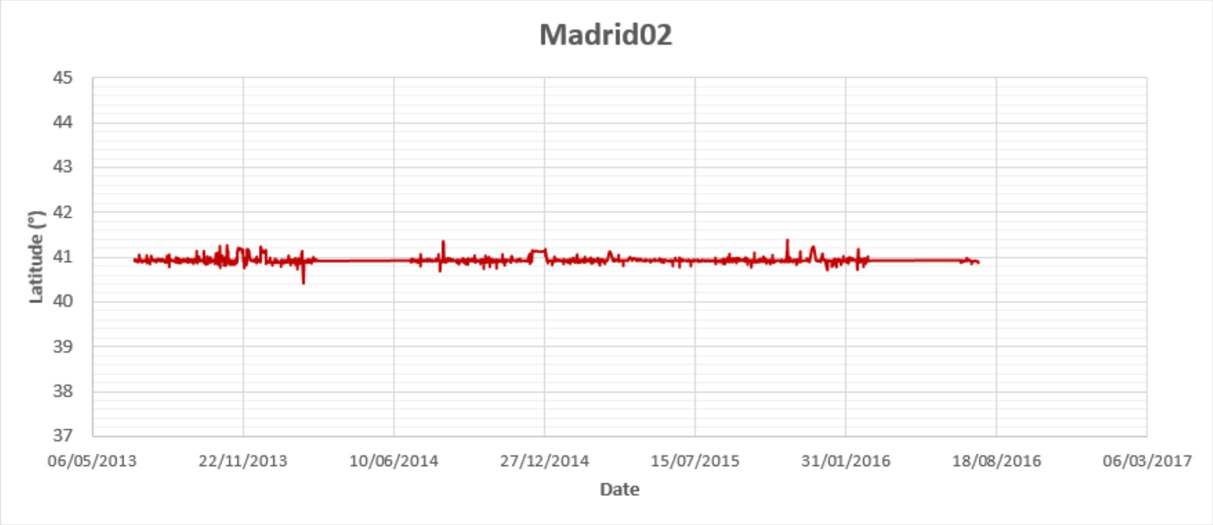
Madrid02
45
44
43
42
41
40
39
38
37
Latitude (°)
06/05/2013
22/11/2013
10/06/2014
27/12/2014
15/07/2015
31/01/2016
18/08/2016
06/03/2017
Date

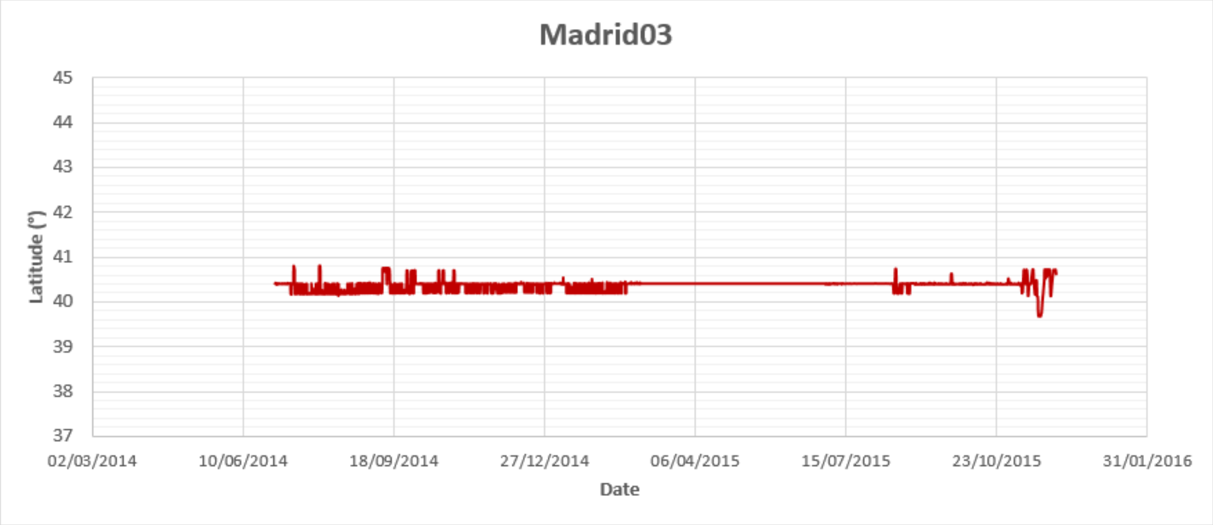
Madrid03
45
44
43
42
41
40
39
38
37
Latitude (°)
02/03/2014
10/06/2014
18/09/2014
27/12/2014
06/04/2015
15/07/2015
23/10/2015
31/01/2016
Date

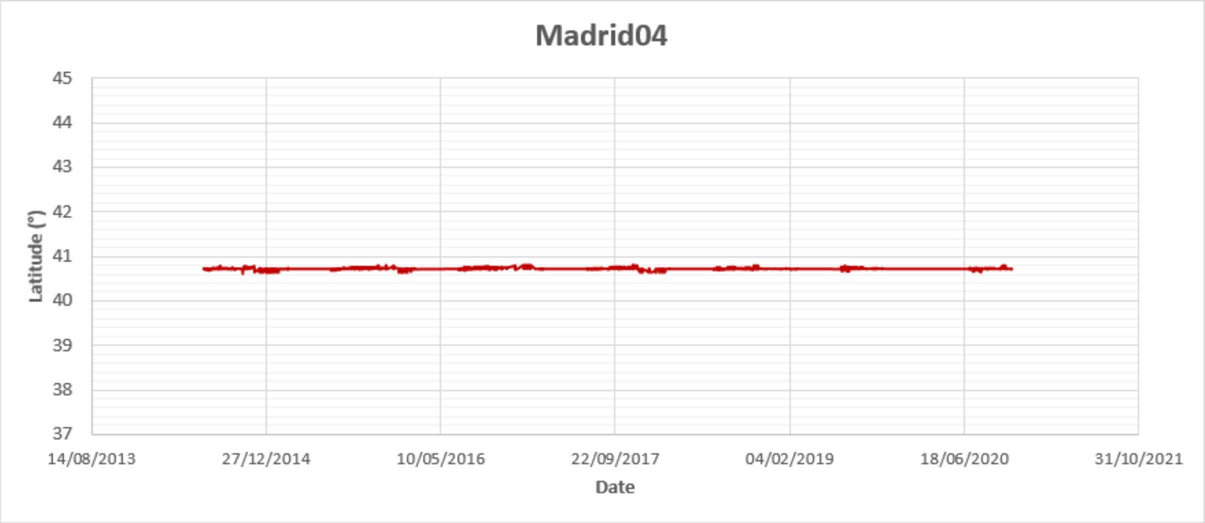
Madrid04
45
44
43
42
41
40
39
38
37
Latitude (°)
14/08/2013
27/12/2014
10/05/2016
22/09/2017
04/02/2019
18/06/2020
31/10/2021
Date

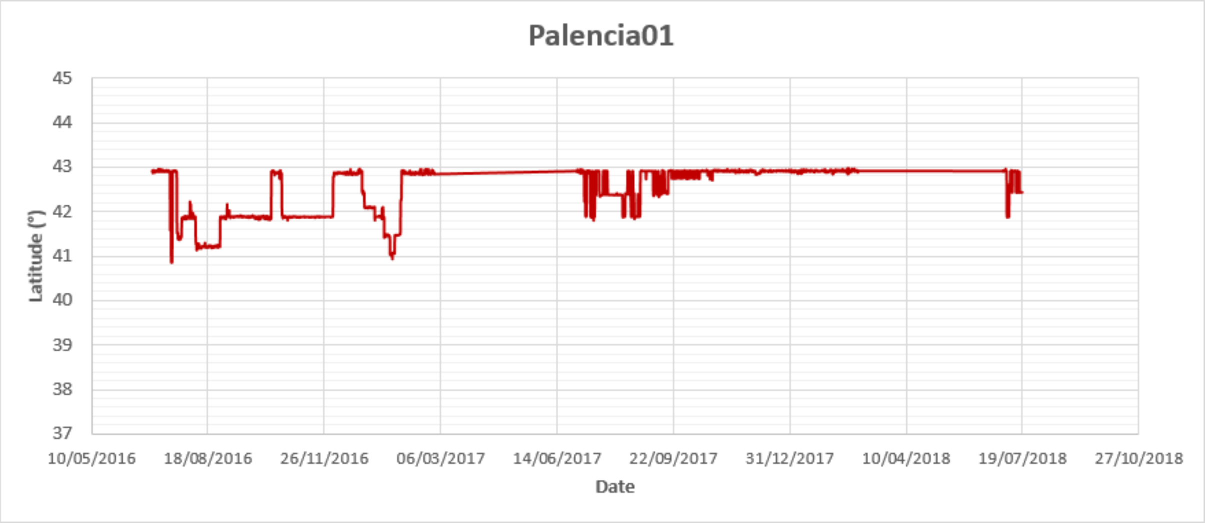
Palencia01
45
44
43
42
41
40
39
38
37
Latitude (°)
10/05/2016
18/08/2016
26/11/2016
06/03/2017
14/06/2017
22/09/2017
31/12/2017
10/04/2018
19/07/2018
27/10/2018
Date

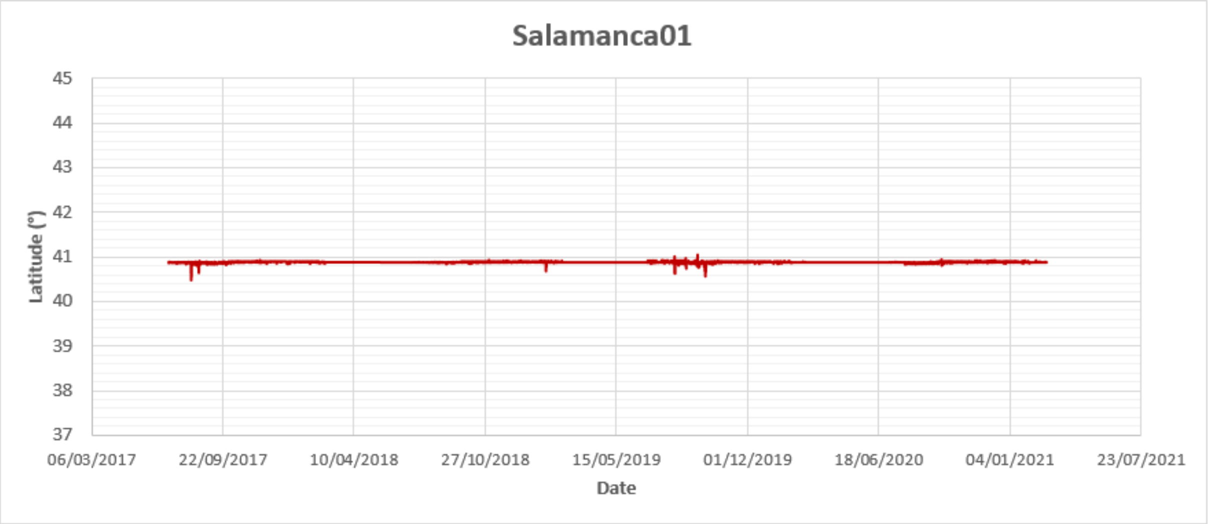
Salamanca01
45
44
43
42
41
40
39
38
37
Latitude (°)
06/03/2017
22/09/2017
10/04/2018
27/10/2018
15/05/2019
01/12/2019
18/06/2020
04/01/2021
23/07/2021
Date

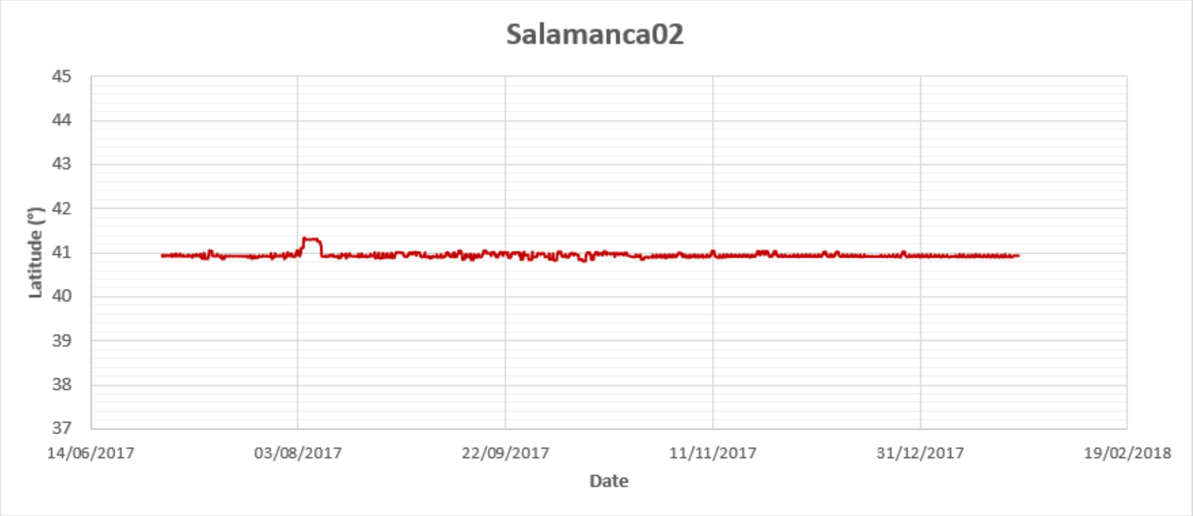
Salamanca02
45
44
43
42
41
40
39
38
37
Latitude (°)
14/06/2017
03/08/2017
22/09/2017
11/11/2017
31/12/2017
19/02/2018
Date

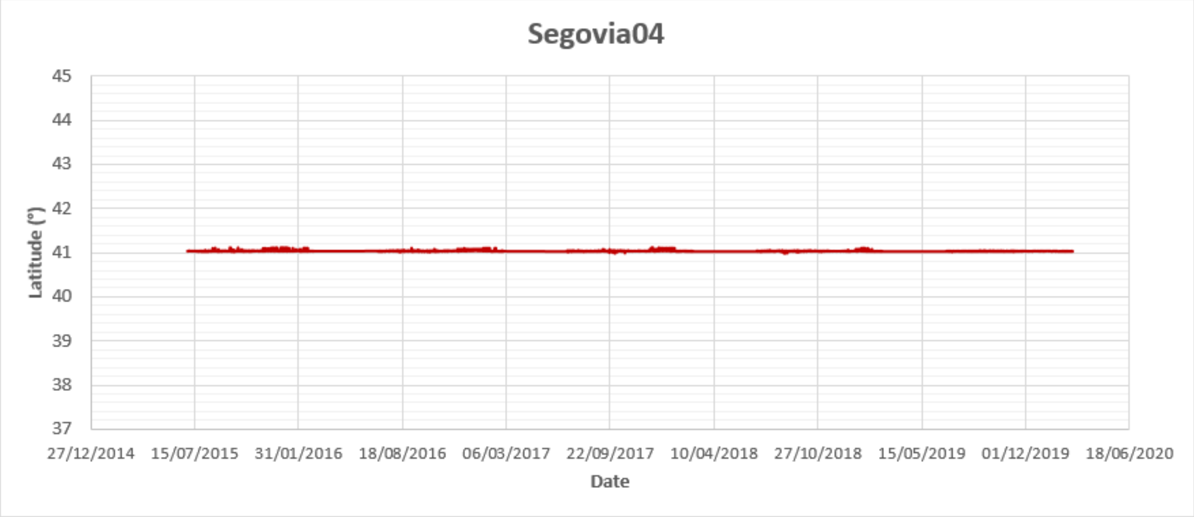
Segovia04
45
44
43
42
41
40
39
38
37
Latitude (°)
27/12/2014
15/07/2015
31/01/2016
18/08/2016
06/03/2017
22/09/2017
10/04/2018
27/10/2018
15/05/2019
01/12/2019
18/06/2020
Date

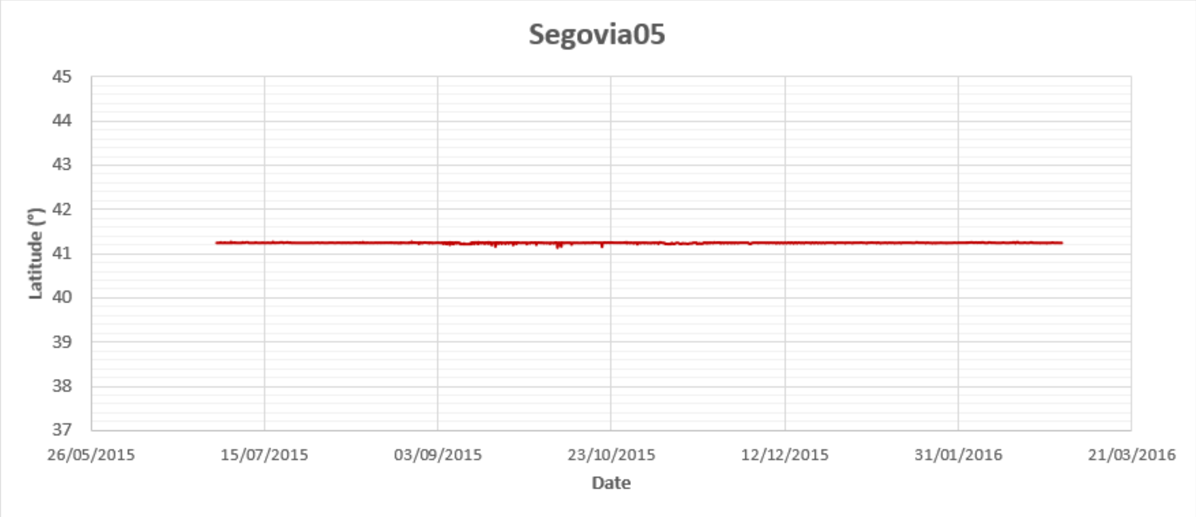
Segovia05
45
44
43
42
41
40
39
38
37
Latitude (°)
26/05/2015
15/07/2015
03/09/2015
23/10/2015
12/12/2015
31/01/2016
21/03/2016
Date

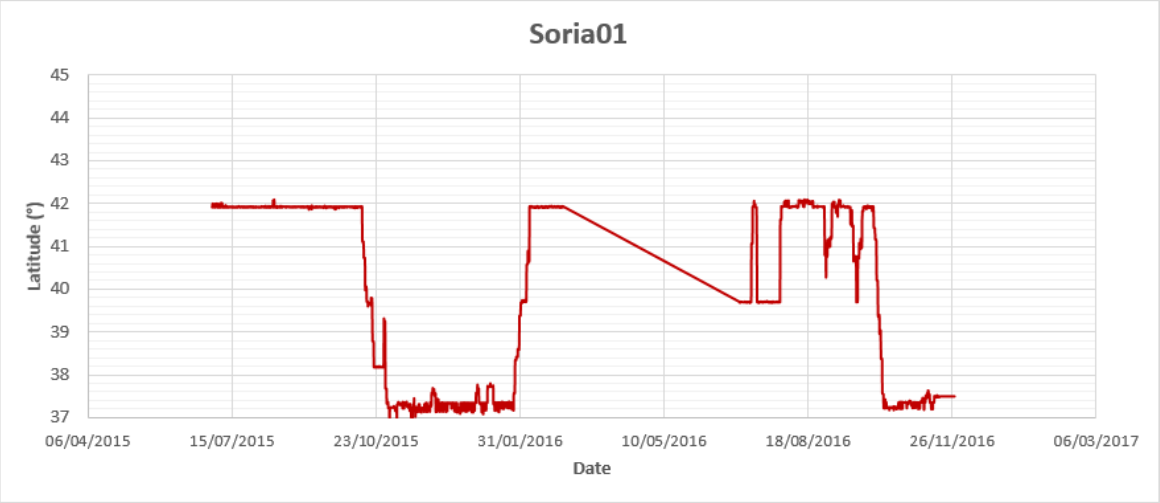
Soria01
45
44
43
42
41
40
39
38
37
Latitude (°)
06/04/2015
15/07/2015
23/10/2015
31/01/2016
10/05/2016
18/08/2016
26/11/2016
06/03/2017
Date

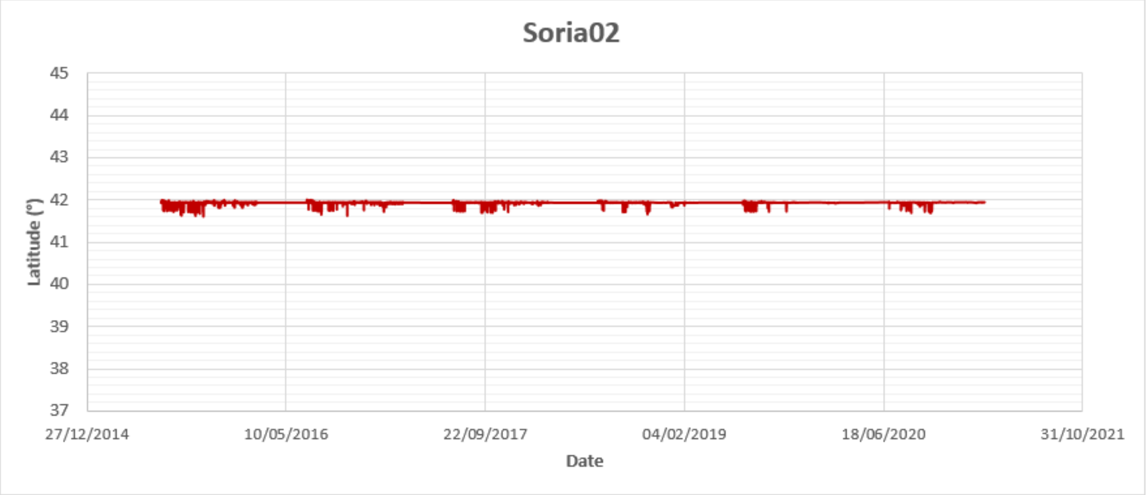
Soria02
45
44
43
42
41
40
39
38
37
Latitude (°)
27/12/2014
10/05/2016
22/09/2017
04/02/2019
18/06/2020
31/10/2021
Date

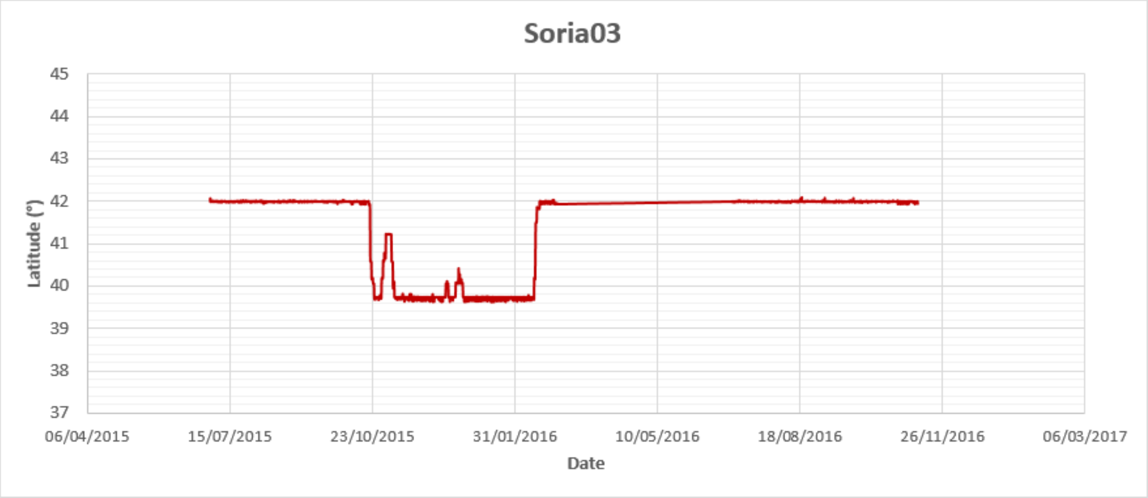
Soria03
45
44
43
42
41
40
39
38
37
Latitude (°)
06/04/2015
15/07/2015
23/10/2015
31/01/2016
10/05/2016
18/08/2016
26/11/2016
06/03/2017
Date

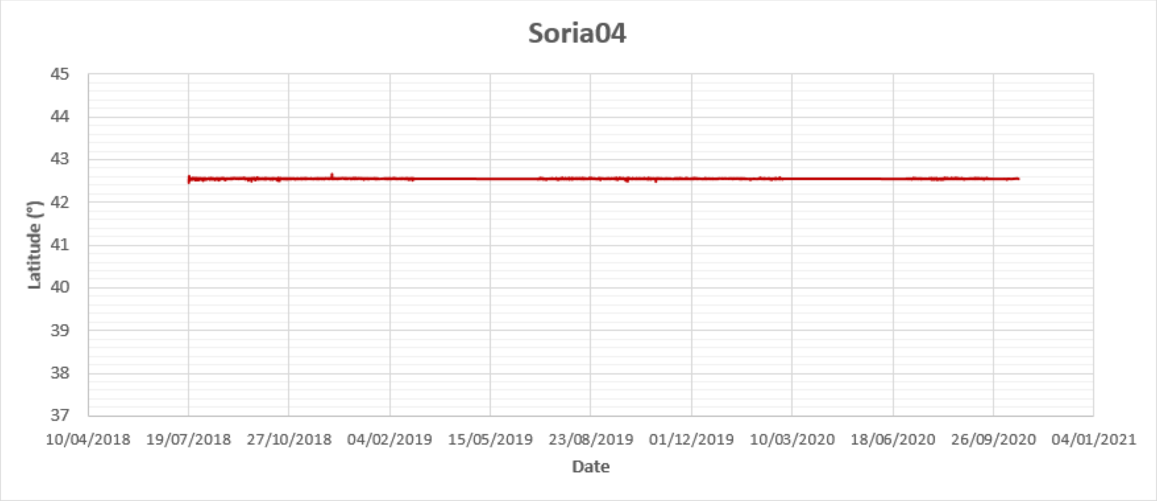
Soria04
45
44
43
42
41
40
39
38
37
Latitude (°)
10/04/2018
19/07/2018
27/10/2018
04/02/2019
15/05/2019
23/08/2019
01/12/2019
10/03/2020
18/06/2020
26/09/2020
04/01/2021
Date

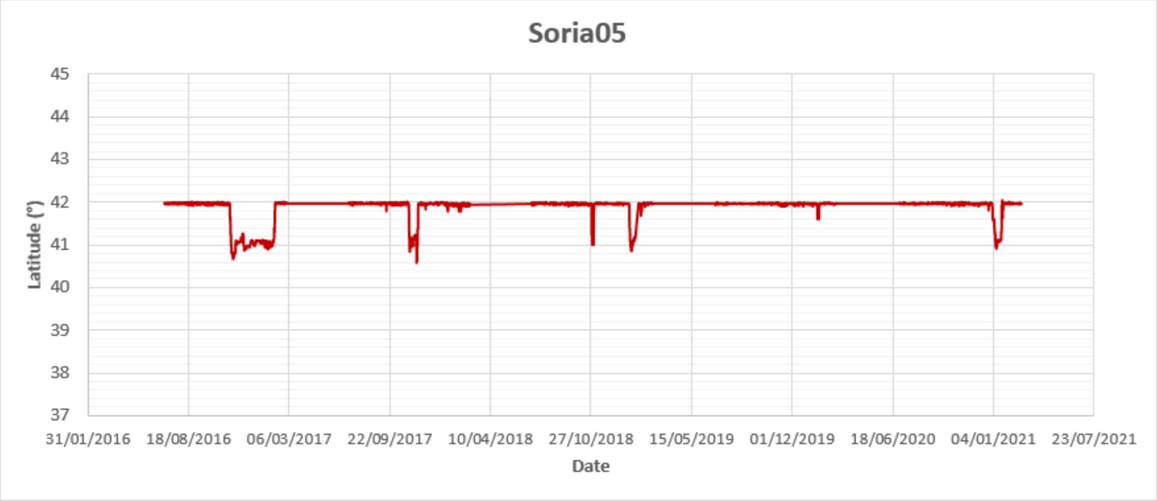
Soria05
45
44
43
42
41
40
39
38
37
Latitude (°)
31/01/2016
18/08/2016
06/03/2017
22/09/2017
10/04/2018
27/10/2018
15/05/2019
01/12/2019
18/06/2020
04/01/2021
23/07/2021
Date

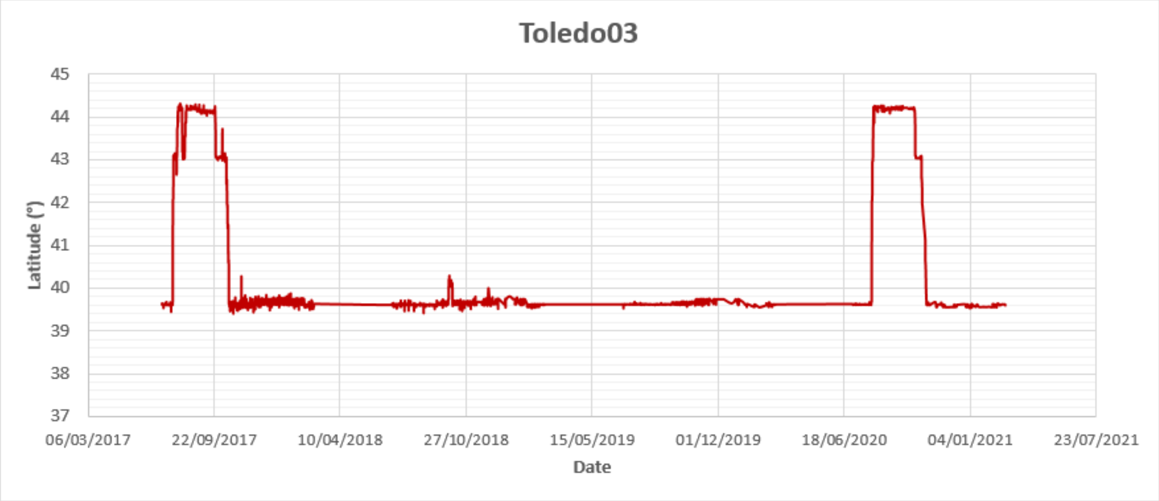
Toledo03
45
44
43
42
41
40
39
38
37
Latitude (°)
06/03/2017
22/09/2017
10/04/2018
27/10/2018
15/05/2019
01/12/2019
18/06/2020
04/01/2021
23/07/2021
Date

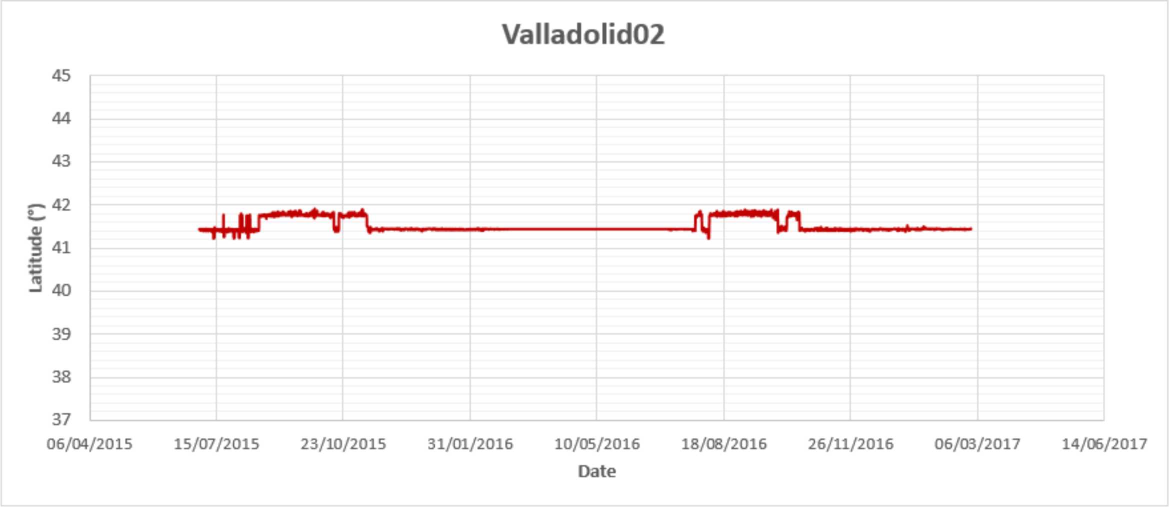
Valladolid02
45
44
43
42
41
40
39
38
37
Latitude (°)
06/04/2015
15/07/2015
23/10/2015
31/01/2016
10/05/2016
18/08/2016
26/11/2016
06/03/2017
14/06/2017
Date

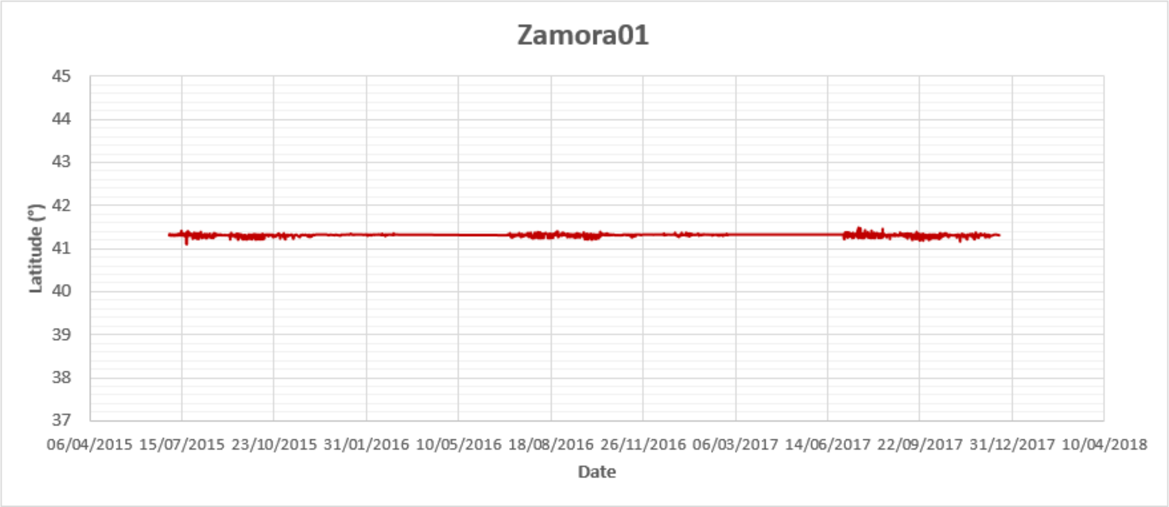
Zamora01
45
44
43
42
41
40
39
38
37
Latitude (°)
06/04/2015
15/07/2015
23/10/2015
31/01/2016
10/05/2016
18/08/2016
26/11/2016
06/03/2017
14/06/2017
22/09/2017
31/12/2017
10/04/2018
Date

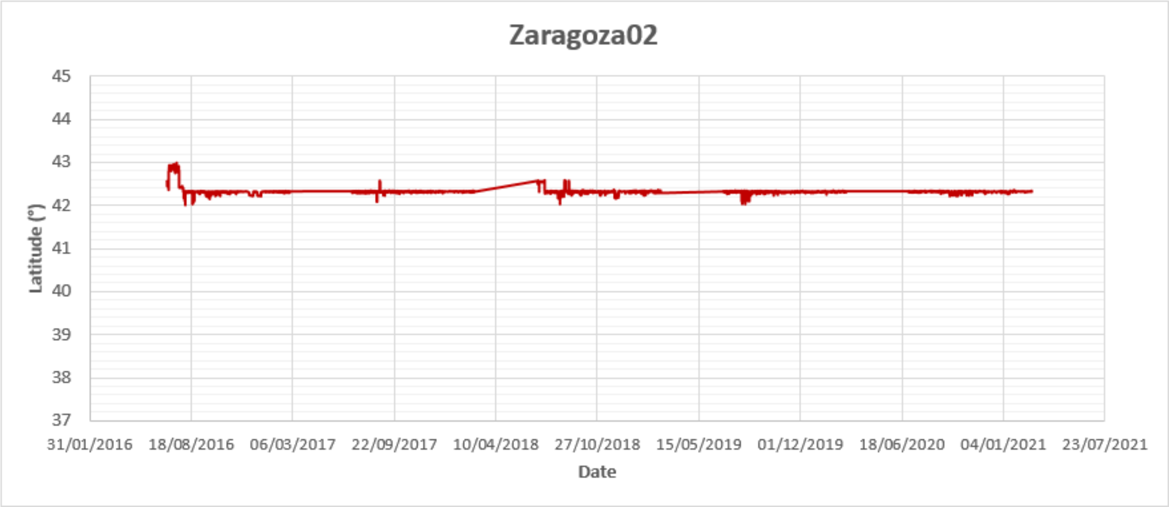
Zaragoza02
45
44
43
42
41
40
39
38
37
Latitude (°)
31/01/2016
18/08/2016
06/03/2017
22/09/2017
10/04/2018
27/10/2018
15/05/2019
01/12/2019
18/06/2020
04/01/2021
23/07/2021
Date

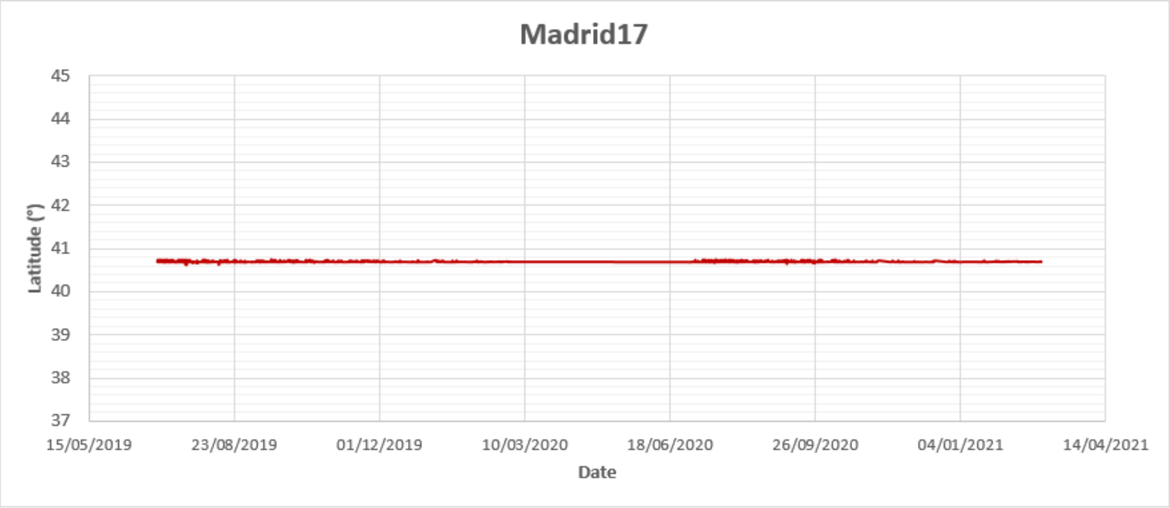
Madrid17
45
44
43
42
41
40
39
38
37
Latitude (°)
15/05/2019
23/08/2019
01/12/2019
10/03/2020
18/06/2020
26/09/2020
04/01/2021
14/04/2021
Date

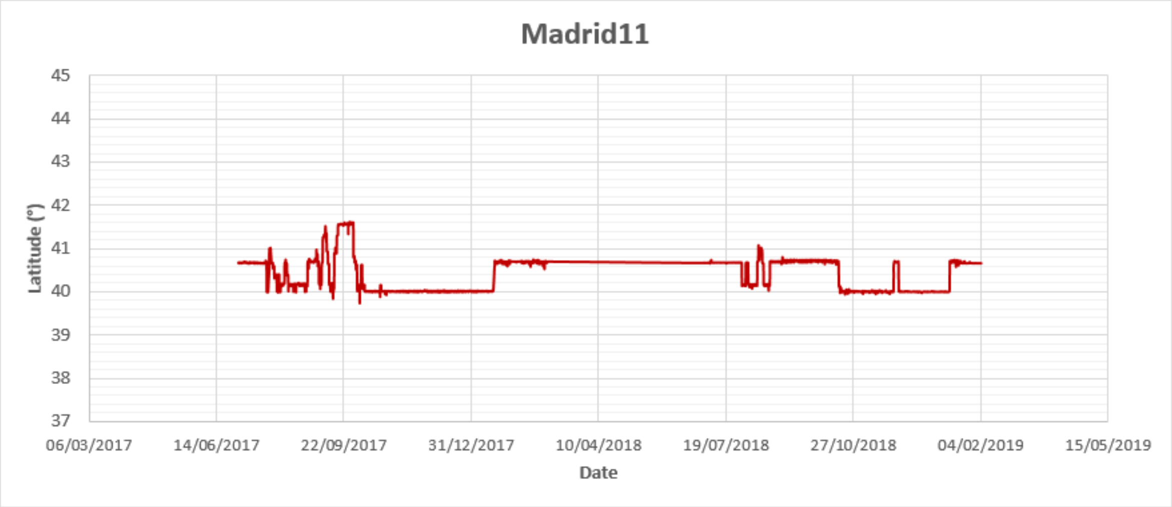
Madrid11
45
44
43
42
41
40
39
38
37
Latitude (°)
06/03/2017
14/06/2017
22/09/2017
31/12/2017
10/04/2018
19/07/2018
27/10/2018
04/02/2019
15/05/2019
Date

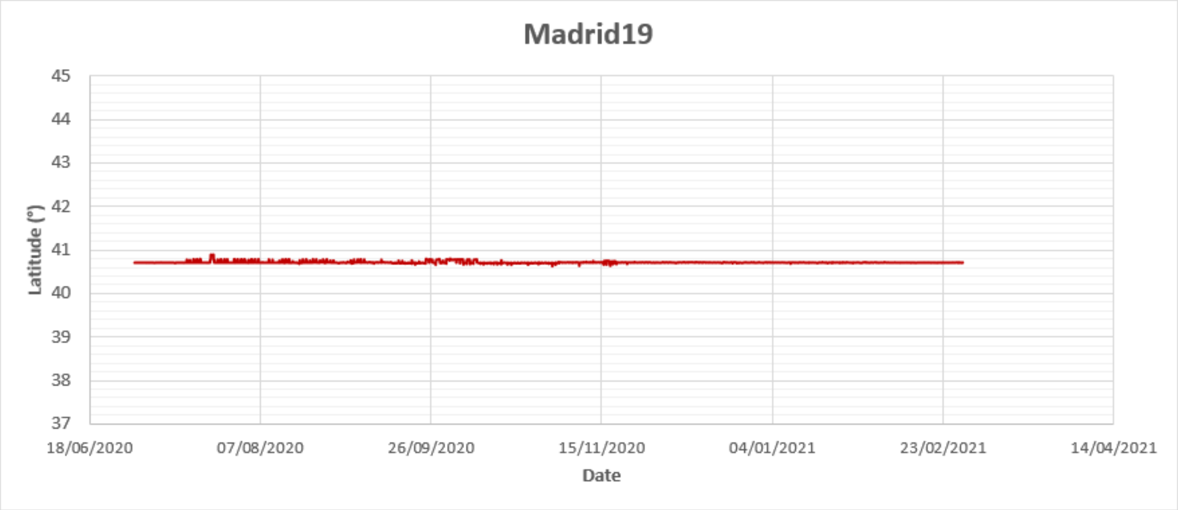
Madrid19
45
44
43
42
41
40
39
38
37
Latitude (°)
18/06/2020
07/08/2020
26/09/2020
15/11/2020
04/01/2021
23/02/2021
14/04/2021
Date

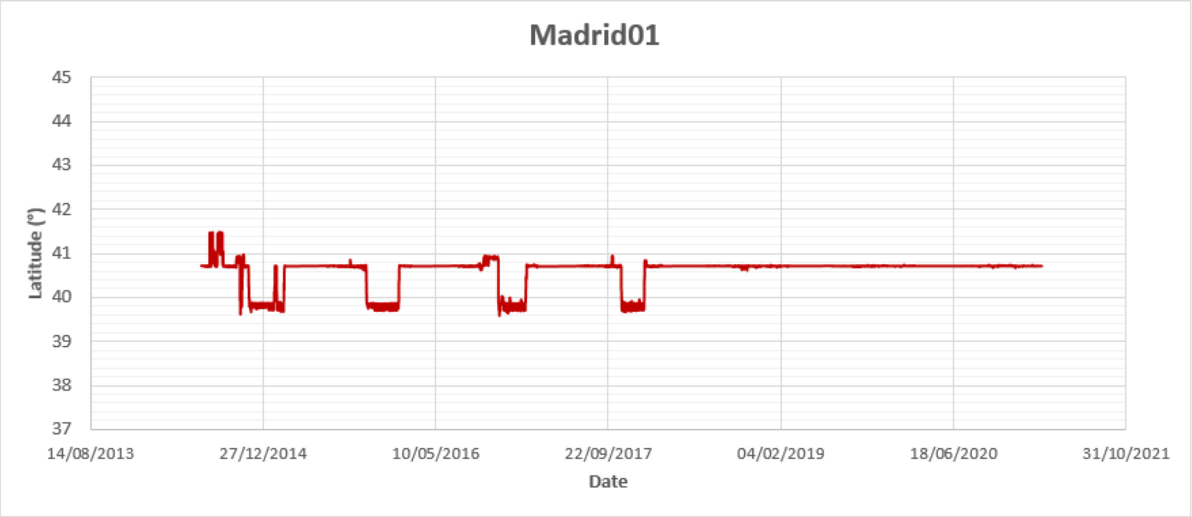
Madrid01
45
44
43
42
41
40
39
38
37
Latitude (°)
14/08/2013
27/12/2014
10/05/2016
22/09/2017
04/02/2019
18/06/2020
31/10/2021
Date

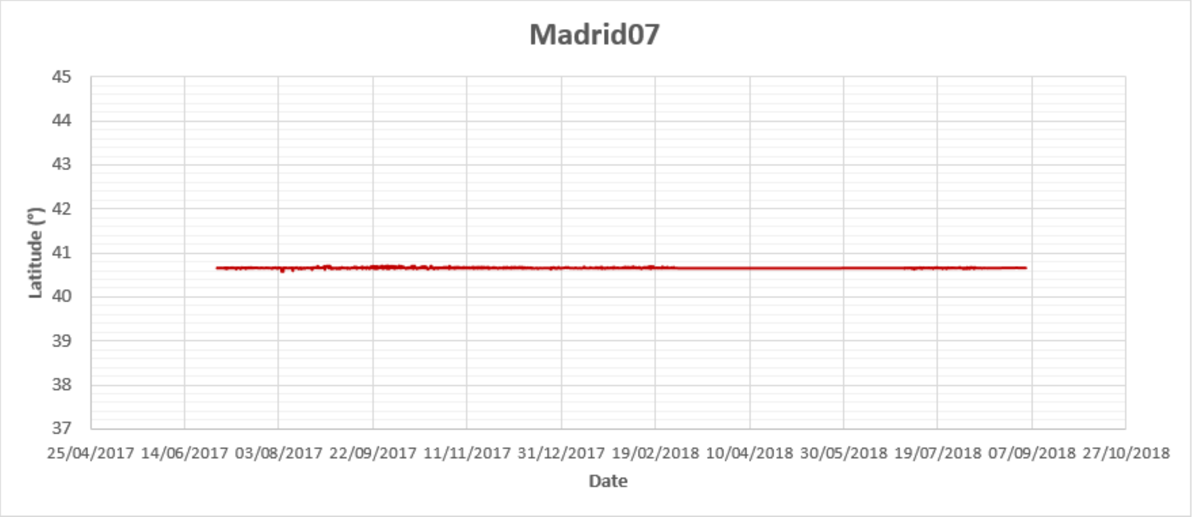
Madrid07
45
44
43
42
41
40
39
38
37
Latitude (°)
25/04/2017
14/06/2017
03/08/2017
22/09/2017
11/11/2017
31/12/2017
19/02/2018
10/04/2018
30/05/2018
19/07/2018
07/09/2018
27/10/2018
Date

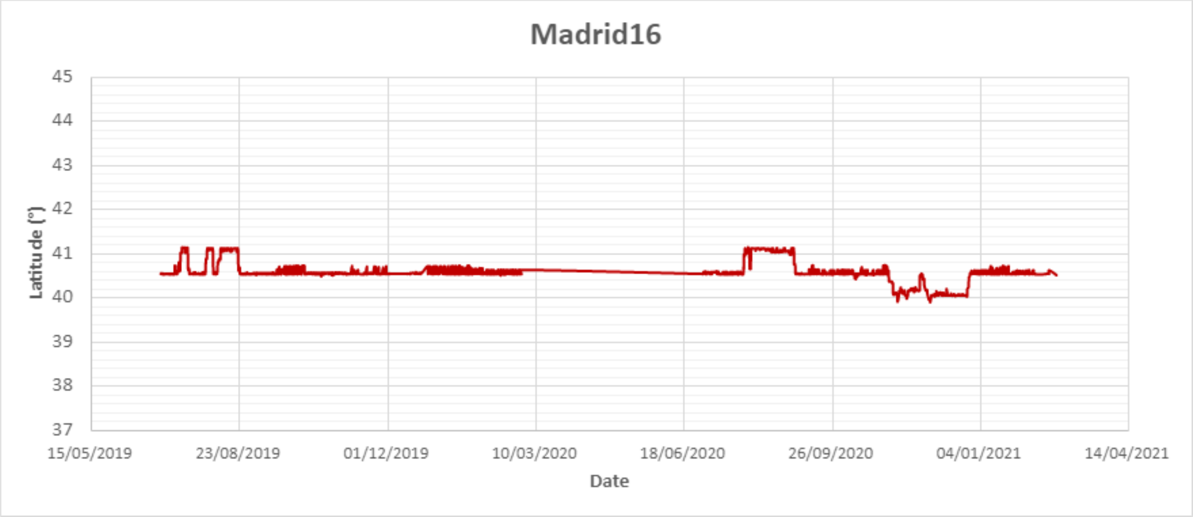
Madrid16
45
44
43
42
41
40
39
38
37
Latitude (°)
15/05/2019
23/08/2019
01/12/2019
10/03/2020
18/06/2020
26/09/2020
04/01/2021
14/04/2021
Date

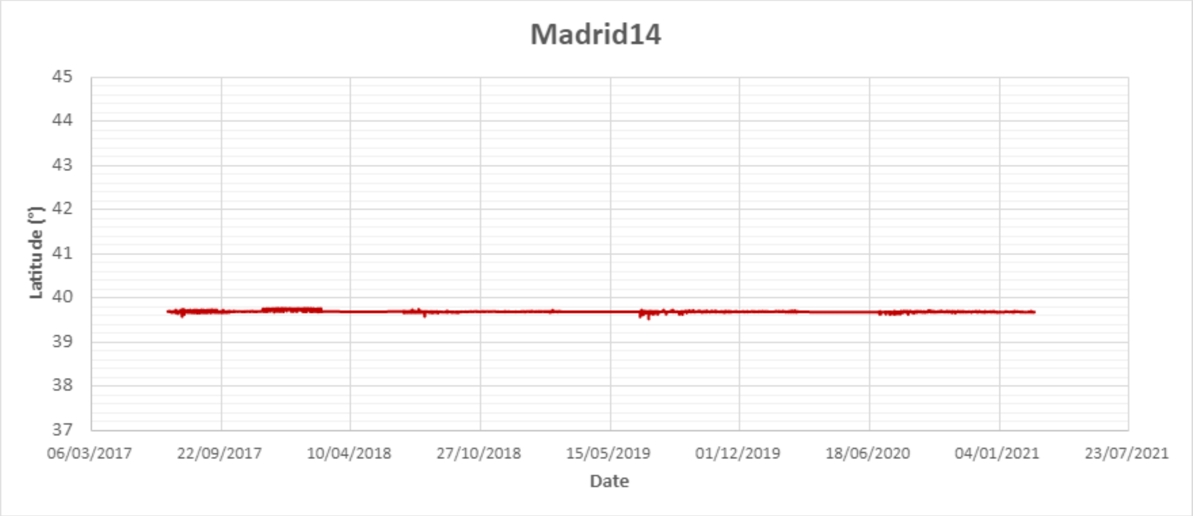
Madrid14
45
44
43
42
41
40
39
38
37
Latitude (°)
06/03/2017
22/09/2017
10/04/2018
27/10/2018
15/05/2019
01/12/2019
18/06/2020
04/01/2021
23/07/2021
Date

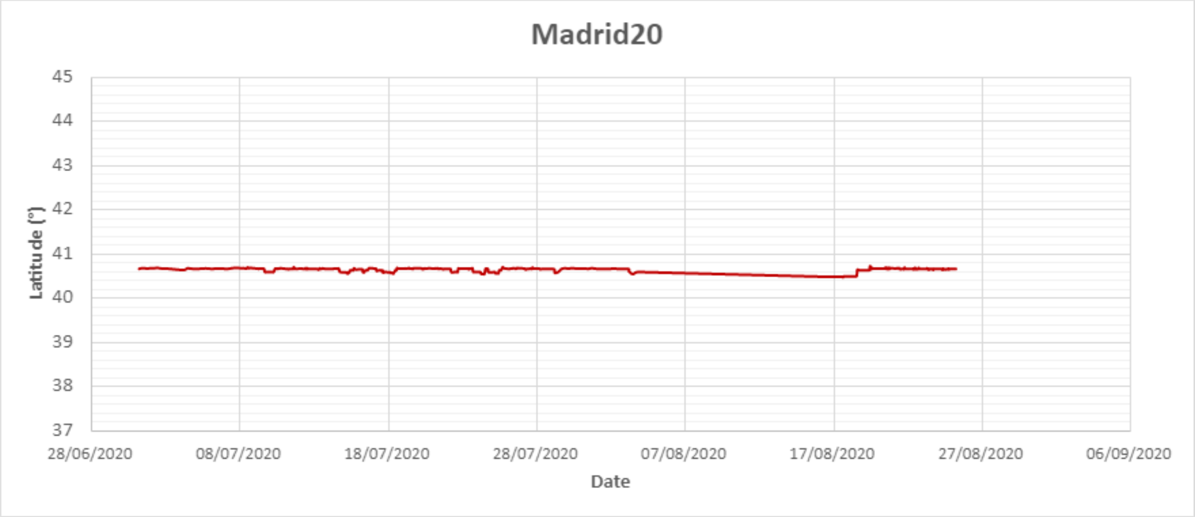
Madrid20
45
44
43
42
41
40
39
38
37
Latitude (°)
28/06/2020
08/07/2020
18/07/2020
28/07/2020
07/08/2020
17/08/2020
27/08/2020
06/09/2020
Date

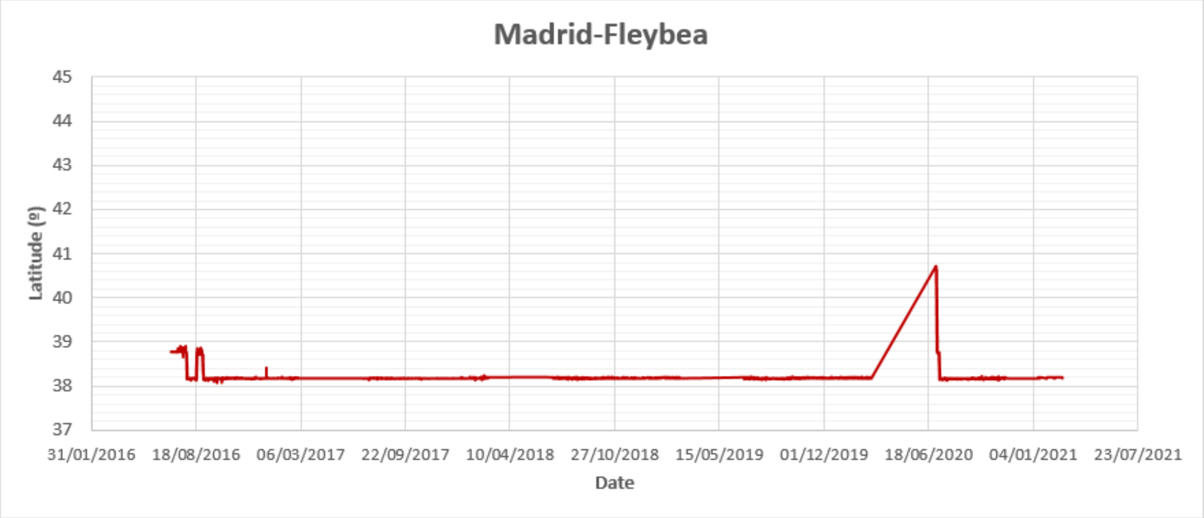
Madrid-Fleybea
45
44
43
42
41
40
39
38
37
Latitude (º)
31/01/2016
18/08/2016
06/03/2017
22/09/2017
10/04/2018
27/10/2018
15/05/2019
01/12/2019
18/06/2020
04/01/2021
23/07/2021
Date

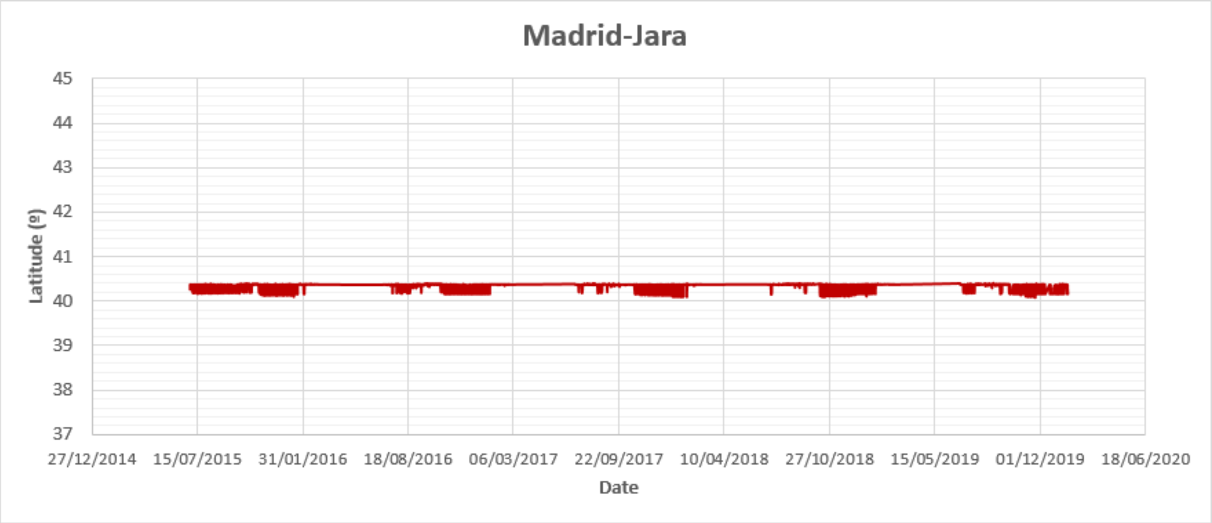
Madrid-Jara
45
44
43
42
41
40
39
38
37
Latitude (º)
27/12/2014
15/07/2015
31/01/2016
18/08/2016
06/03/2017
22/09/2017
10/04/2018
27/10/2018
15/05/2019
01/12/2019
18/06/2020
Date

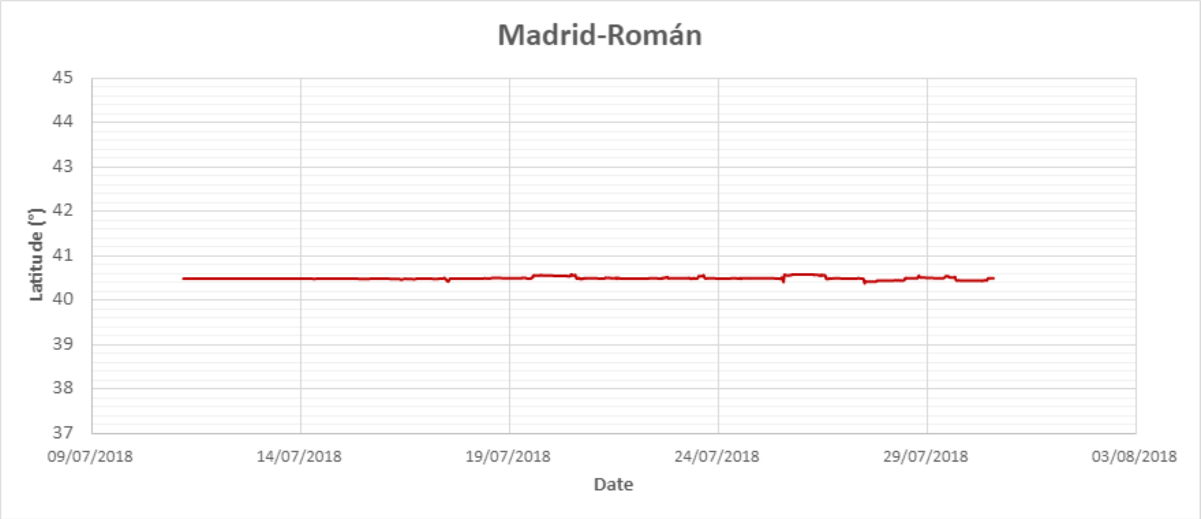
Madrid-Román
45
44
43
42
41
40
39
38
37
Latitude (°)
09/07/2018
14/07/2018
19/07/2018
24/07/2018
29/07/2018
03/08/2018
Date

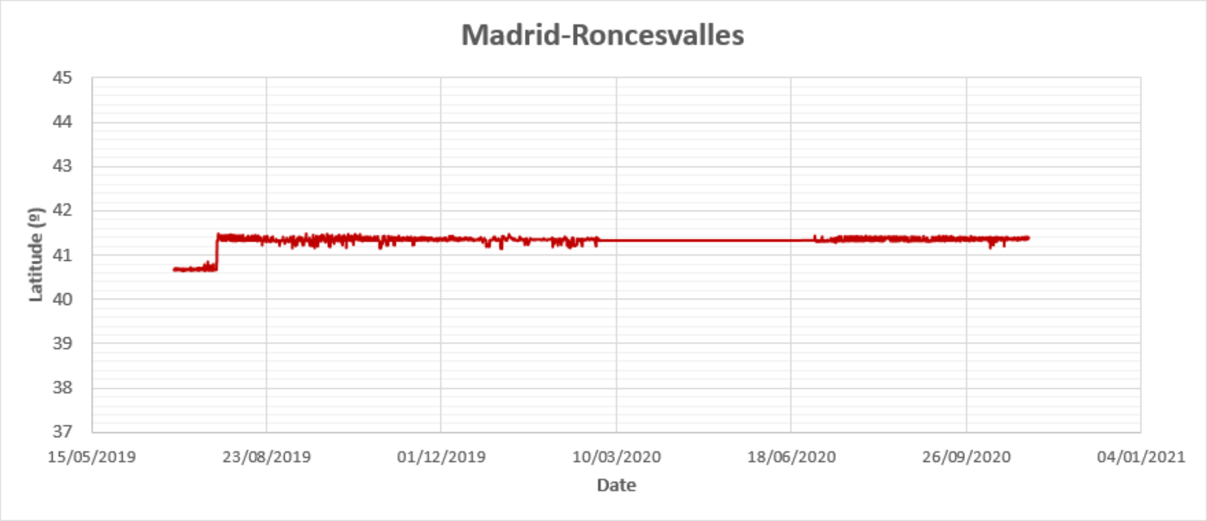
Madrid-Roncesvalles
45
44
43
42
41
40
39
38
37
Latitude (º)
15/05/2019
23/08/2019
01/12/2019
10/03/2020
18/06/2020
26/09/2020
04/01/2021
Date

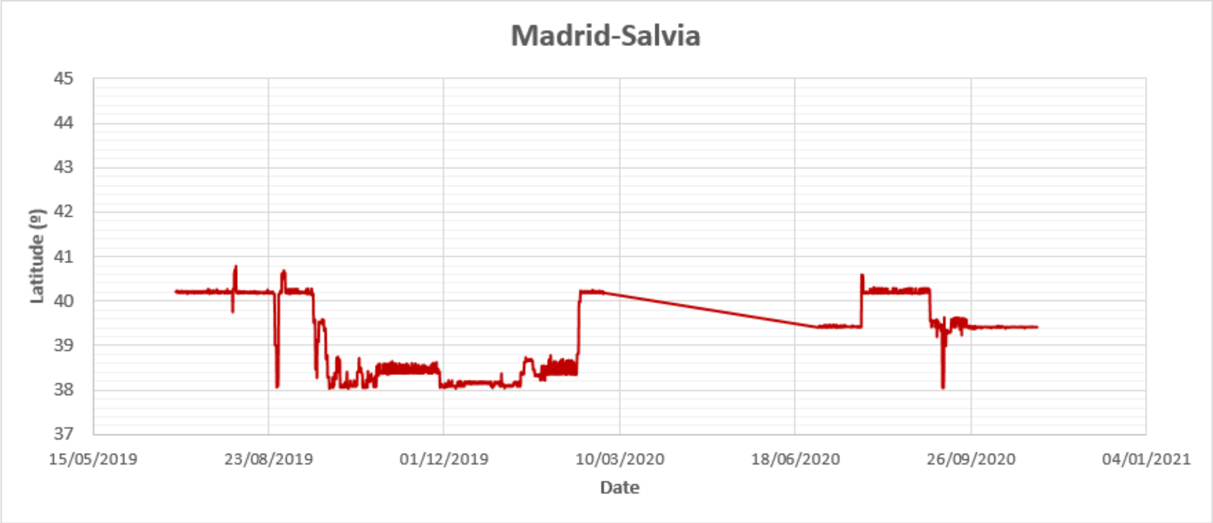

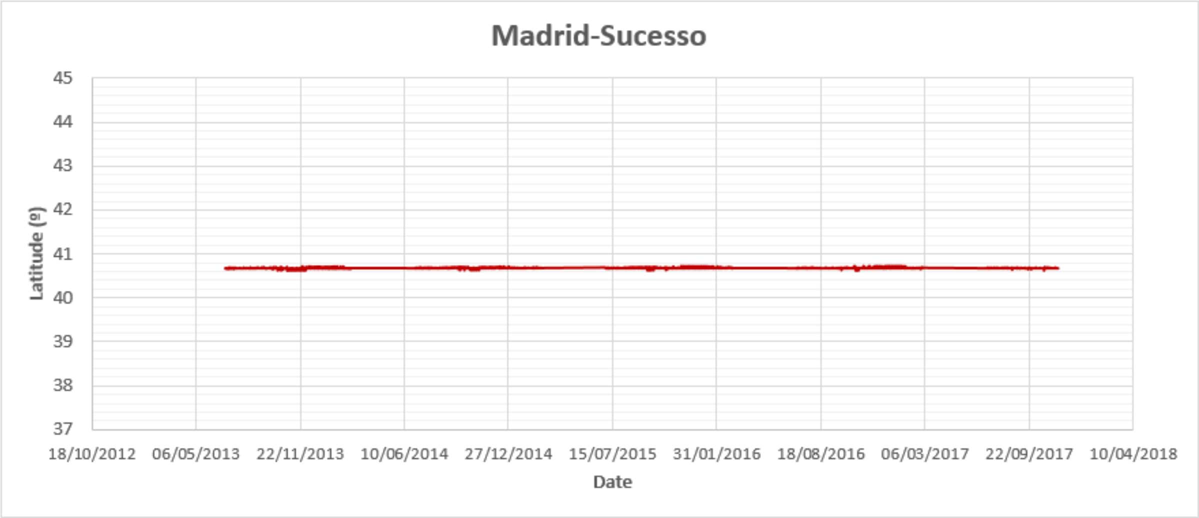

Figure S2. Latitudinal variation of breeding Red Kites tagged in Spain. Some sedentary with post-reproductive movements may be under-represented because they also had a marked longitudinal component. The breeding period considered (March-June) is not represented, so a straight line joins the last location of February and the first location of June.
